# Supplementary material for: Design and protocol of a comprehensive multicentre biobank for abdominal aortic aneurysms
Source: BMJ Open. 2019 Aug 1;9(8):e028858. doi: 10.1136/bmjopen-2018-028858 (PMC6688677; doi:10.1136/bmjopen-2018-028858)
Supplement: Supplementary data [file bmjopen-2018-028858supp001.pdf]

English translation of the informed consent form of the biobank Pearl AAA

Appendix 1. Informed consent form for patients who have an asymptomatic AAA or who have previously undergone AAA repair

Biobank Pearl AAA.

- I consent to the collection, storage and analysis of blood, urine and aortic tissue (in case of open surgery) and data regarding research for abdominal aortic aneurysms.

- I consent to the coded storage of my data for a maximum of 50 years in the biobank Pearl AAA.

- I consent to the coded storage of my biomaterials for a maximum of 50 years for use in future biomedical research.

- I give permission for my GP to be informed that I am participating in this study.

- I give permission for information regarding my health status to be requested from my GP, pharmacy and the municipal registry of persons, under the condition that my privacy will be safeguarded.

- During biomedical research with my biomaterials, incidental findings may be found that are associated with a disease or disorder. Sometimes it only concerns an increased risk. I consent that I will be informed about incidental findings if these are relevant to my (or my family's) health status because either prevention or treatment is possible.

- I consent to the possible sharing of my data, biomaterials and imaging data with non-commercial institutions in countries in the European Union. This will only be done for research purposes.

Additional consent options:

- I consent to the acquisition of my data from national registries concerning the quality of healthcare and its improvement, under the condition that my privacy will be safeguarded.
    - Yes
    - No

- In the event of my death, I consent to the possibility that information regarding my death may be acquired from the Central Bureau of Statistics, under the condition that my privacy will be safeguarded.
    - Yes
    - No

- I consent to the possible sharing of my data, biomaterials and imaging data with institutions in countries outside the European Union. This will only be done for research purposes.
    - Yes
    - No

- I consent to the possible sharing of my data, biomaterials and imaging data with commercial entities. This will only be done for research purposes.
    - Yes

   - No

- I consent to the study of my DNA for findings associated with the origin and development of abdominal aortic aneurysms and to the effects and complications of medication
   - Yes
   - No

- I consent that I may be approached for additional information and/or biomaterials if this is necessary for a specific biomedical study
   - Yes
   - No

Name participant:
Date of birth:
Signature: Date: __/ __/ __

Declaration of researcher

I hereby declare that I have fully informed this study participant regarding the biobank Pearl AAA. If information comes to light during the course of the study that could affect the study subject's consent, I will inform him/her of this in a timely fashion.

Name researcher:
Signature: Date: __/ __/ __

Additional information has been given by (if applicable):
Name:
Role:
Signature: Date: __/ __/ __

Study participants receive the full information brochure, and a signed copy of the informed consent form.
