# Supplementary material for: Design and protocol of a comprehensive multicentre biobank for abdominal aortic aneurysms
Source: BMJ Open. 2019 Aug 1;9(8):e028858. doi: 10.1136/bmjopen-2018-028858 (PMC6688677; doi:10.1136/bmjopen-2018-028858)
Supplement: Supplementary data [file bmjopen-2018-028858supp002.pdf]

English translation of the informed consent form of the PARIS study

Appendix 2. Informed consent form for patients who have an asymptomatic AAA or who have previously undergone AAA repair

PARIS study

- I have read the subject information form. I was also able to ask questions. My questions have been answered to my satisfaction. I had enough time to decide whether to participate.

- I know that participation is voluntary. I know that I may decide at any time not to participate after all or to withdraw from the study. I do not need to give a reason for this.

- I give permission for my GP to be informed that I am participating in this study.

- I give permission for information to be requested from my GP or pharmacy regarding my health status.

- I know that some people may have access to all my data to verify the study. These people are listed in this information sheet. I consent to the inspection by them.

- I consent to the collection and use of my data, biomaterials and imaging data as has been specified in this information sheet.

- I consent to the storage of my data for the PARIS study for a maximum of 50 years in the biobank Pearl AAA.

- I consent to the possible sharing of my data, biomaterials and imaging data with non-commercial institutions in countries in the European Union. This will only be done for the PARIS study if necessary.

Additional consent options:

- I consent to the possible sharing of my data, biomaterials and imaging data with institutions in countries outside the European Union. This will only be done for the PARIS study if necessary.
    - Yes
    - No

- I consent to the possible sharing of my data, biomaterials and imaging data with commercial entities. This will only be done for the PARIS study if necessary.
    - Yes
    - No

- I consent to being contacted again after this study for a follow-up study.
    - Yes
    - No

- I want to participate in this study.

English translation of the informed consent form of the PARIS study

Name participant:
Date of birth:
Signature: Date: __/ __/ __

Declaration of researcher

I hereby declare that I have fully informed this study participant regarding the PARIS study. If information comes to light during the course of the study that could affect the study subject's consent, I will inform him/her of this in a timely fashion.

Name researcher:
Signature: Date: __/ __/ __

Additional information has been given by (if applicable):
Name:
Role:
Signature: Date: __/ __/ __

Study participants receive the full information brochure, and a signed copy of the informed consent form.
