# Supplementary material for: Design and protocol of a comprehensive multicentre biobank for abdominal aortic aneurysms
Source: BMJ Open. 2019 Aug 1;9(8):e028858. doi: 10.1136/bmjopen-2018-028858 (PMC6688677; doi:10.1136/bmjopen-2018-028858)
Supplement: Supplementary data [file bmjopen-2018-028858supp003.pdf]

English translation of the informed consent form of the biobank Pearl AAA

Appendix 3. Informed consent form for patients after emergency AAA repair.

Biobank Pearl AAA

- I consent to the collection, storage and analysis of blood, urine and aortic tissue (in case of open surgery) and data regarding research for abdominal aortic aneurysms.

- I consent to the coded storage of my data for a maximum of 50 years in the biobank Pearl AAA.

- I consent to the coded storage of my biomaterials for a maximum of 50 years for use in future biomedical research.

- I give permission that my GP to be informed that I am participating in this study.

- I give permission for information regarding my health status to be requested from my GP, pharmacy and the municipal registry of persons, under the condition that my privacy will be safeguarded.

- During biomedical research with my biomaterials, incidental findings may be found that are associated with a disease or disorder. Sometimes it only concerns an increased risk. I consent that I will be informed about incidental findings if these are relevant to my (or my family's) health status because either prevention or treatment is possible.

- I consent to the possible sharing of my data, biomaterials and imaging data with non-commercial institutions in countries in the European Union. This will only be done for research purposes.

- I consent to the acquisition of my data from national registries concerning the quality of healthcare and its improvement, under the condition that my privacy will be safeguarded.
    - Yes
    - No

- In the event of my death, I consent to the possibility that information regarding my death may be acquired from the Central Bureau of Statistics, under the condition that my privacy will be safeguarded.
    - Yes
    - No

- I consent to the possible sharing of my data, biomaterials and imaging data with institutions in countries outside the European Union. This will only be done for research purposes.
    - Yes
    - No

- I consent to the possible sharing of my data, biomaterials and imaging data with commercial entities. This will only be done for research purposes.
    - Yes
    - No

English translation of the informed consent form of the biobank Pearl AAA

- I consent to the study of my DNA for findings associated with the origin and development of abdominal aortic aneurysms and to the effects and complications of medication
    - Yes
    - No

- I consent that I may be approached for additional information and/or biomaterials if this is necessary for a specific biomedical study
    - Yes
    - No

Name participant:
Date of birth:
Signature: Date: __/ __/ __

Declaration of researcher

I hereby declare that I have fully informed the legal representative regarding the biobank Pearl AAA. If information comes to light during the course of the study that could affect the legal representative's consent, I will inform him/her of this in a timely fashion.

Name researcher:
Signature: Date: __/ __/ __

Additional information has been given by (if applicable):
Name:
Role:
Signature: Date: __/ __/ __

Study participants receive the full information brochure, and a signed copy of the informed consent form.
