# Supplementary material for: Design and protocol of a comprehensive multicentre biobank for abdominal aortic aneurysms
Source: BMJ Open. 2019 Aug 1;9(8):e028858. doi: 10.1136/bmjopen-2018-028858 (PMC6688677; doi:10.1136/bmjopen-2018-028858)
Supplement: Supplementary data [file bmjopen-2018-028858supp005.pdf]

English translation of the informed consent form of the biobank Pearl AAA

Appendix 5. Informed consent form for legal representative.

Biobank Pearl AAA

I have been asked to give written consent for the participation of the following person in the biobank Parel AAA.

Name person:
Date of birth: __/ __/ __

- I consent to the collection, storage and analysis of blood, urine and aortic tissue (in case of open surgery) and data of the person regarding research for abdominal aortic aneurysms.
- I consent to the coded storage of the data for a maximum of 50 years in the biobank Pearl AAA.
- I consent to the coded storage of the biomaterials for a maximum of 50 years for use in future biomedical research.
- I give permission that the GP of the person is to be informed that the person is participating in this study.
- I give permission for information regarding the health status of the person to be requested from the GP and pharmacy, under the condition that his/her privacy will be safeguarded.
- During biomedical research with the biomaterials, incidental findings may be found that are associated with a disease or disorder. Sometimes it only concerns an increased risk. I consent that the GP of the person will be informed about incidental findings if these are relevant to the family's health status because either prevention or treatment is possible.
- I consent to the possible sharing of the data, biomaterials and imaging data with non-commercial institutions in countries the European Union. This will only be done for research purposes.

Additional consent options:

- I consent to the acquisition of data regarding the person from national registries concerning the quality of healthcare and its improvement, under the condition that his/her privacy will be safeguarded.
    - Yes
    - No
- I consent to the possible sharing of the data, biomaterials and imaging data of the person with institutions in countries outside the European Union. This will only be done for research purposes.
    - Yes
    - No
- I consent to the possible sharing of the data, biomaterials and imaging data with commercial entities. This will only be done for research purposes.

  - Yes
  - No

- I consent to the study of the DNA for findings associated with the origin and development of abdominal aortic aneurysms and to the effects and complications of medication
  - Yes
  - No

Name legal representative:
Date of birth:
Relation to the person:
Signature: Date: __/ __/ __

Declaration of researcher

I hereby declare that I have fully informed the legal representative regarding the biobank Pearl AAA. If information comes to light during the course of the study that could affect the legal representative's consent, I will inform him/her of this in a timely fashion.

Name researcher:
Signature: Date: __/ __/ __

Additional information has been given by (if applicable):
Name:
Role:
Signature: Date: __/ __/ __

Study participants receive the full information brochure, and a signed copy of the informed consent form.
