# Supplementary material for: Design and protocol of a comprehensive multicentre biobank for abdominal aortic aneurysms
Source: BMJ Open. 2019 Aug 1;9(8):e028858. doi: 10.1136/bmjopen-2018-028858 (PMC6688677; doi:10.1136/bmjopen-2018-028858)
Supplement: Supplementary data [file bmjopen-2018-028858supp006.pdf]

English translation of the informed consent form of the PARIS study

Appendix 6. Informed consent form for legal representative.

PARIS study

I have been asked to give written consent for the participation of the following person in the PARIS study.

Name person:
Date of birth: __/ __/ __

- I have read the subject information form. I was also able to ask questions. My questions have been answered to my satisfaction. I had enough time to decide whether this person participates.

- I know that participation is voluntary. I know that I may decide at any time that this person does not to participate after all or to withdraw him/her from the study. I do not need to give a reason for this.

- I give permission for the GP of this person to be informed that he/she is participating in this study.

- I give permission for information to be requested from the GP or pharmacy of this person regarding his/her health status.

- I know that some people may have access to all this person's data to verify the study. These people are listed in this information sheet. I consent to the inspection by them.

- I consent to the collection and use of the data, biomaterials and imaging data as has been specified in this information sheet.

- I consent to the storage of the data for the PARIS study for a maximum of 50 years in the biobank Pearl AAA.

- I consent to the possible sharing of the data, biomaterials and imaging data with non-commercial institutions in countries in the European Union. This will only be for the PARIS study if necessary.

- I consent to the possible sharing of the data, biomaterials and imaging data with institutions in countries outside the European Union. This will only be done for the PARIS study.
    - Yes
    - No

- I consent to the possible sharing of the data, biomaterials and imaging data with commercial entities. This will only be done for the PARIS study.
    - Yes
    - No

- I agree with the participation of this person with the study.

English translation of the informed consent form of the PARIS study

Name legal representative:
Date of birth:
Relation to the person:
Signature: Date: __/ __/ __

Declaration of researcher

I hereby declare that I have fully informed the legal representative regarding the biobank Pearl AAA. If information comes to light during the course of the study that could affect the legal representative's consent, I will inform him/her of this in a timely fashion.

Name researcher:
Signature: Date: __/ __/ __

Additional information has been given by (if applicable):
Name:
Role:
Signature: Date: __/ __/ __

Study participants receive the full information brochure, and a signed copy of the informed consent form.
