# Supplementary material for: Design and protocol of a comprehensive multicentre biobank for abdominal aortic aneurysms
Source: BMJ Open. 2019 Aug 1;9(8):e028858. doi: 10.1136/bmjopen-2018-028858 (PMC6688677; doi:10.1136/bmjopen-2018-028858)
Supplement: Author's manuscript [file bmjopen-2018-028858.draft_revisions.pdf]

BMJ Open is committed to open peer review. As part of this commitment we make the peer review history of every article we publish publicly available.

When an article is published we post the peer reviewers' comments and the authors' responses online. We also post the versions of the paper that were used during peer review. These are the versions that the peer review comments apply to.

The versions of the paper that follow are the versions that were submitted during the peer review process. They are not the versions of record or the final published versions. They should not be cited or distributed as the published version of this manuscript.

BMJ Open is an open access journal and the full, final, typeset and author-corrected version of record of the manuscript is available on our site with no access controls, subscription charges or pay-per-view fees (http://bmjopen.bmj.com).

If you have any questions on BMJ Open's open peer review process please email info.bmjopen@bmj.com

BMJ Open

Design and protocol of a comprehensive multicentre biobank for abdominal aortic aneurysms

Journal:	*BMJ Open*
Manuscript ID	bmjopen-2018-028858
Article Type:	Protocol
Date Submitted by the Author:	02-Jan-2019
Complete List of Authors:	Jalalzadeh, Hamid; Amsterdam UMC, Surgery<br>Indrakusuma, Reza; Amsterdam UMC, Surgery<br>Blankensteijn, Jan; Amsterdam UMC, Surgery<br>Wisselink, Willem; Amsterdam UMC, Surgery<br>Yeung, Kakkhee; Amsterdam UMC, Surgery<br>Lindeman, Jan; Leiden University Medical Center, Vascular surgery<br>Hamming, Jaap; LUMC, surgery<br>Koelemay, Mark; Amsterdam UMC, Surgery<br>Legemate, Dink; Amsterdam UMC, Surgery<br>Balm, Ron; Amsterdam UMC, Surgery
Keywords:	Vascular surgery < SURGERY, VASCULAR SURGERY, Cardiovascular imaging < RADIOLOGY & IMAGING

SCHOLARONE™
Manuscripts

Design and protocol of a comprehensive multicentre biobank for abdominal aortic aneurysms

Hamid Jalalzadeh*[1], Reza Indrakusuma*[1], Jan D. Blankensteijn[2], Willem Wisselink[2], Kak K. Yeung[2], Jan H.N. Lindeman[3], Jaap F. Hamming[3], Mark J.W. Koelemay[1], Dink A. Legemate[1], Ron Balm[1]

* Both authors contributed equally

1. Amsterdam UMC, University of Amsterdam, Department of Surgery, Amsterdam Cardiovascular Sciences, Meibergdreef 9, 1105 AZ, Amsterdam, The Netherlands
2. Amsterdam UMC, Vrije Universiteit Amsterdam, Department of Surgery, Amsterdam Cardiovascular Sciences, De Boelelaan 1117, 1081 HV, Amsterdam, The Netherlands
3. Leiden University Medical Center, Department of Surgery, Albinusdreef 2, 2333 ZA, Leiden, The Netherlands

Current steering committee:

Hamid Jalalzadeh: h.jalalzadeh@amc.uva.nl

Reza Indrakusuma: r.indrakusuma@amc.uva.nl

Jan D. Blankensteijn: j.blankensteijn@vumc.nl

Willem Wisselink: w.wisselink@vumc.nl

Kak. K. Yeung: k.yeung@vumc.nl

Jan H.N. Lindeman: j.h.n.lindeman@lumc.nl

Jaap F. Hamming: j.f.hamming@lumc.nl

Mark. J.W. Koelemay: m.j.koelemaij@amc.uva.nl

Dink A. Legemate: d.a.legemate@amc.uva.nl

Ron Balm: r.balm@amc.nl

For peer review only

Corresponding author and sponsor-investigator:

Ron Balm

r.balm@amc.nl

telephone number: +31205667832

Amsterdam UMC, University of Amsterdam, Department of Surgery, Amsterdam Cardiovascular

Sciences, Meibergdreef 9, 1105 AZ, Amsterdam, The Netherlands

Licence per instructions

I, the Submitting Author has the right to grant and does grant on behalf of all authors of the Work (as defined in the below author licence), an exclusive licence and/or a non-exclusive licence for contributions from authors who are: i) UK Crown employees; ii) where BMJ has agreed a CC-BY licence shall apply, and/or iii) in accordance with the terms applicable for US Federal Government officers or employees acting as part of their official duties; on a worldwide, perpetual, irrevocable, royalty-free basis to BMJ Publishing Group Ltd ("BMJ") its licensees and where the relevant Journal is co-owned by BMJ to the co-owners of the Journal, to publish the Work in BMJ Open and any other BMJ products and to exploit all rights, as set out in our licence.

The Submitting Author accepts and understands that any supply made under these terms is made by BMJ to the Submitting Author unless you are acting as an employee on behalf of your employer or a postgraduate student of an affiliated institution which is paying any applicable article publishing charge ("APC") for Open Access articles. Where the Submitting Author wishes to make the Work available on an Open Access basis (and intends to pay the relevant APC), the terms of reuse of such Open Access shall be governed by a Creative Commons licence – details of these licences and which Creative Commons licence will apply to this Work are set out in our licence referred to above.

Word count:

- Abstract: 328
- Manuscript excluding title page, abstract, tables, acknowledgements, contributions and references: 2457

For peer review only

ABSTRACT

Introduction

The pathophysiology and natural course of abdominal aortic aneurysms (AAAs) are insufficiently understood. In order to improve our understanding, it is imperative to carry out longitudinal research which combines biomarkers with data and imaging markers, all measured over multiple time points. Therefore, a multicentre biobank, databank and imagebank has been established in the Netherlands: the '*Pearl Abdominal Aortic Aneurysm*' (AAA bank).

Methods and analysis

The AAA bank is a prospective multicentre observational bio-, data- and imagebank of patients with an AAA. It is embedded within the framework of the Dutch String of Pearls Institute (PSI) which facilitates uniform biobanking in all university medical centres (UMCs) in the Netherlands. The AAA bank has been initiated by the two UMCs of Amsterdam UMC and by Leiden University Medical Center. Included patients with AAA will be followed during AAA follow-up. At every patient contact, clinical data is collected for standardised storage in the databank. Three types of biomaterials are collected at baseline and during follow-up: blood (including DNA and RNA), urine and AAA tissue if open surgical repair is performed. Imaging data that are obtained as part of clinical care are stored in the imagebank. All data and biomaterials are processed and stored in a standardised manner. AAA growth will be based on multiple measurements and will therefore be analysed with a repeated measures analysis. Potential associations between AAA growth and risk factors that are also measured on multiple time points can be assessed with a multivariate mixed-effects model, while potential associations between AAA rupture and risk factors can be tested with a conditional dynamic prediction model with landmarking.

Ethics and dissemination

The AAA bank is approved by the Medical ethics Board of the Amsterdam UMC (University of Amsterdam).

For peer review only

Registration: this study was retrospectively registered on October 25, 2017 on www.clinicaltrials.gov (NCT03320408).

Keywords:

Abdominal aortic aneurysm, biobank, databank, imagebank, pathophysiology, natural history

For peer review only

Strengths and limitations of this study

- Longitudinal collection of clinical data, biomaterials and imaging data of patients with an abdominal aortic aneurysm provides ample opportunity to better understand the natural history, and to search for prognostic risk factors that might benefit future treatment.
- The inclusion of patients with a small asymptomatic abdominal aortic aneurysm offers the possibility to study natural history from an early stage.
- Standardised collection and storage of biomaterials allows the analysis of patients included at different hospitals.
- Study follow-up is done at routine follow-up appointments to minimise participant burden, yet also means that study follow-up will vary between patients.
- Patient recruitment currently only takes place in university medical centres which are tertiary referral centres.

For peer review only

INTRODUCTION

An abdominal aortic aneurysm (AAA) is a focal dilatation of the abdominal aorta that affects mostly elderly men. The prevalence of AAA in the general population is 1.3-2.2% in 65-year old men.[1, 2] An AAA is an asymptomatic disorder which is associated with a high risk of mortality in case of rupture.[3] Management of patients with an AAA is aimed at preventing rupture, either by surveillance for small AAAs or by prophylactic AAA repair if the rupture risk is deemed high, in general at a diameter of more than 5.5 cm. A large body of research has been dedicated to determining the optimal surgical treatment for AAA, focusing on either the threshold diameter for repair, the method of repair, or the outcome and follow-up after treatment. Although many studies have tried to unravel AAA pathophysiology, this aspect is still insufficiently understood. Most of the current knowledge originates from histopathological studies that reflect the end stage of AAA disease. Early determinants and drivers of AAA formation are largely unidentified due to the unavailability of AAA tissue from an early stage of the disease. Therefore, the known determinants of AAA development are limited to general risk factors such as male sex, ageing, smoking or connective tissue diseases.[4, 5]

Recent studies focused on biomarker research to find early disease markers and potential targets for pharmacological treatment. Promising circulating biomarkers included markers of matrix turnover (matrix metalloproteinases), markers of inflammation (interleukins and C-reactive protein) and markers of lipid metabolism (lipoproteins).[6-8] Unfortunately, to date, none of these biomarkers have found their way to clinical practice. This is mostly due to their low prognostic value for AAA progression, and because many studies have not corrected for factors as smoking or comorbidities.[7] The same applies to new imaging biomarkers such as ^{18}F-fluoro-deoxy-glucose (^{18}F-FDG), or biomechanical markers such as peak wall stress and wall shear stress.[9-11]

To better understand AAA pathophysiology and its natural course, it is imperative to combine studies with biomarkers, imaging markers, and longitudinal data. To that end, a multicentre databank, biobank and imagebank has been established in the Netherlands: the '*Pearl Abdominal Aortic Aneurysm*, hereafter referred to as the AAA bank. The aim of this project is to facilitate future studies

For peer review only

on AAA. It is part of the Dutch String of Pearls Institute (PSI), which facilitates uniform biobanking in all eight university medical centres (UMCs) in the Netherlands.[12] The systematic collection of clinical data, biomaterials and imaging data will enable a diverse range of studies on patients with AAA. The AAA bank will especially focus on patients with a small AAA to collect longitudinal data early on in the development of AAA.

The first proposed study that will be carried out with the collected biomaterials is the 'Predicting aneurysm growth and rupture with longitudinal biomarkers' (PARIS) study. The PARIS study aims to determine the association between AAA progression and the evolution of serum levels of proteases and cytokines.

The scientific aims of the AAA bank are (1) to gain insight in the pathophysiology and natural history of AAA; (2) to gain more knowledge about the rupture risk of AAA; and (3) to evaluate and improve treatment of patients with an AAA. Future studies with data from the AAA bank must adhere to these scientific aims.

For peer review only

METHODS AND ANALYSIS

Study design

The AAA bank is a prospective multicentre observational biobank, databank and imagebank of patients with AAA in The Netherlands (ClinicalTrials.gov: NCT03320408, see table 1 for the WHO Trial Registration Data Set).

Table 1. WHO trial registration data set

Primary registry and trial identifying number	ClinicalTrials.gov: NCT03320408
Date of registration in primary registry	25 October 2017
Secondary identifying numbers	NL59991.018.17, PARIS study, biobank Pearl AAA
Sources of monetary of material support	AMC Foundation for monetary support.
Primary sponsor	Academic Medical Center – University of Amsterdam
Secondary sponsor(s)	none
Contact for public queries	Els Kuiters, e.kuiters@amc.uva.nl, +31205667832, Meibergdreef 9, 1105AZ, Amsterdam, the Netherlands.
Contact for scientific queries	Principal investigator: prof. R. Balm, r.balm@amc.nl, +3120-5667832, Meibergdreef 9, 1105AZ, Amsterdam, The Netherlands.<br><br>Els Kuiters, e.kuiters@amc.uva.nl, +31205667832, Meibergdreef 9, 1105AZ, Amsterdam, the Netherlands.
Public title	PARIS study & biobank Pearl AAA
Scientific title	Predicting aneurysm growth and rupture with longitudinal biomarkers (PARIS study) & biobank Pearl AAA
Countries of recruitment	The Netherlands
Health condition(s)	Abdominal aortic aneurysm
Intervention(s)	None
Key inclusion and exclusion criteria	Inclusion criteria: adult with an AAA, or who has been previously treated for an AAA. Adequate comprehension of the Dutch language to provide written informed consent.<br>Exclusion criteria: Decisionally impaired patients. The exception are patients who are decisionally impaired due to the effects of an acute AAA for whom a separate recruitment and consent procedure exists.
Study type	Observational longitudinal patient registry and biobank
Date of first enrolment	4 October 2017
Sample size	Planned: 750<br>Currently enrolled: 87
Recruitment status	Recruiting; participants are currently being recruited and enrolled
Primary outcome(s)	Outcome: AAA growth. Time frame: up to 10 years of follow-up<br>Outcome. AAA rupture. Time frame: up to 10 years of follow-up<br>Outcome: all-cause mortality. Time frame: up to 10 years of follow-up<br>Outcome: evolution of serum levels of proteases and cytokines. Time frame:

For peer review only

	a maximum of 1 measurement annually up to 10 years of follow-up<br>Outcome: proteases and cytokine levels in AAA tissue. Time frame: if open AAA repair is performed and AAA tissue is collected. This is a one-time measurement.
Key secondary outcome(s)	Outcome: incidence and type of complications after AAA repair. Time frame: up to 10 years of follow-up after AAA repair
Ethics review	Status: approved<br>Date of approval: 25 August 2017<br>Name and contact details of ethics committees:<br>Medical Ethics Board of Amsterdam UMC (University of Amsterdam). mecamc@amc.uva.nl, +31205667389, Trinity building C, fourth floor, Pietersbergweg 17, 1105BM Amsterdam, the Netherlands.<br><br>Biobank Ethics Board of Amsterdam UMC (University of Amsterdam), biobanktoetsing@amc.uva.nl, +31205666730, Meibergdreef 9, 1105AZ, Amsterdam, the Netherlands.
Completion date	Expected: 4 October 2032
Summary results	No results yet.
IPD sharing statement	Plan to share IPD: Yes<br>Plan description: IPD sharing for research will be allowed in a data sharing procedure. Scientific requests need to fall under the scope of the scientific aims as formulated in this manuscript. Researchers willing to requests IPD can initiate this procedure by contacting the researchers. Data will be released depending on the scientific quality of the submitted request.

AAA = abdominal aortic aneurysm

The active protocol at the time of writing is version 3, 22 December 2017. The AAA bank is embedded within the framework of PSI, which is co-financed by the Dutch government and the Netherlands Federation of University Medical centres (NFU).[12] PSI was established in 2007 and aims to facilitate standardised nationwide biobanks and clinical databases.[13] At the time of writing, 17 different patient cohorts (*Pearls*) for different diseases have been initiated within the PSI framework. Examples include the Diabetes Pearl, the Pearl Neurodegenerative Diseases, the Stroke Pearl, and the Dutch Pancreas biobank.[14-17] All these biobanks adhere to an internal regulatory framework, which prescribes legal and ethical rules concerning the conduct of all Pearl-related activities.[13]

The AAA bank has been initiated by the two UMCs from Amsterdam University Medical Centers (University of Amsterdam and Vrije Universiteit Amsterdam) and by Leiden University Medical Center (LUMC). In accordance with the goals of PSI and NFU, the AAA bank aims to expand to the

For peer review only

other Dutch UMCs. The AAA bank is currently an ongoing project, with active recruitment and collection of data and biomaterials. The first patient was recruited on October 4, 2017.

Study population

All capable adults with AAA in participating university medical centres are eligible for inclusion in the AAA bank. This also includes patients who previously have undergone AAA repair. Patients who are incapable due to a ruptured AAA (RAAA) can also be included, using a special recruitment process, which will be described below. All included patients will be followed for as long as they visit their treating vascular surgeon.

Recruitment procedure

Eligible patients are recruited at the inpatient or outpatient clinic of the department of Vascular Surgery of the participating hospitals by research physicians and/or data managers. Participants who agree to participate give written informed consent during their visit to the inpatient or outpatient clinic. When patients arrive in an emergency setting with a ruptured or symptomatic AAA, oral consent is required from either the patient or a legal representative. This oral consent has to be confirmed in writing at a later stage – either by the patient, or by a representative in case of a fatal outcome. In the event that no written informed consent can be obtained, all data and biomaterials collected for the AAA bank will be destroyed.

Study procedures

The study procedures of the AAA bank are embedded within regular AAA treatment. Clinical data and biomaterials are collected during visits that are already part of clinical treatment, to limit participant burden and improve participant retention. No research visits will be planned for the AAA bank. In addition, only imaging data that is obtained as part of clinical care will be stored in the AAA bank.

For peer review only

In line with regular AAA management, four distinct study phases have been identified: inclusion phase, surveillance phase, surgical phase, and postoperative phase (Figure 1; blue boxes). Within each phase, multiple visits can take place – especially in the surveillance or postoperative phase. These phases can theoretically continue indefinitely, depending on the course of the AAA. With regard to the 'surveillance phase', the Dutch AAA guideline advises that patients visit a vascular surgeon at intervals of two years, one year, or three months, depending on the AAA diameter.[18] Patients can move from the 'surveillance phase' to the 'surgical phase' and subsequently to the 'postoperative phase' (Figure 1).

Clinical data – PRISMA

At every patient contact, clinical data are collected for storage in the databank of the AAA bank by research physicians and/or data managers. All data items have been incorporated in electronic Data Capture Systems (eDCSs). These eDCSs are integrated in an information model called Parelsnoer Repository for Information Specification, Modelling and Architecture (PRISMA, Table 2).

For peer review only

Table 2. The PRISMA information model of the AAA bank

eDCS name	Inclusion			Follow-up[N]			Stream
	Asymptomatic AAA	Operation for acute AAA	Previously operated AAA	Surveillance phase	Surgical phase	Postoperative phase	
Patient information	1			M			
Informed consent	1			M			
Living situation	1			1			
Current aneurysm state	1			1			
First occurrence comorbidity	1			M			
Family history	0..n			M			
Social history	1			M			
Initial aneurysm characteristics	1						
Comorbidity status	1			1			
Medication	1			1			
Intoxications	1			1			
Physical examination	1			1	1		
Blood test results	1			1			
Surveillance	1			1			
Preoperative assessment		1			1		
AAA repair		1			1		
Postoperative admission		1			1		
Postoperative follow-up		1				1	

For peer review only

Biomaterials	1	1	
Cardiovascular events			dt
Other surgical procedures			dt
Malignancies			dt
AAA imaging	0..n		dt
Complications			dt
Imaging data	1	1	dt
	eDCS: electronic Data Capture System;<br>1: indicates that an eDCS is registered once and is overwritten in case of future changes.<br>M: Means 'Modify', and indicates that the eDCS can be modified within the same observation period. N: Indicates that this eDCS can be registered multiple times (but at least once)<br>0..n: Indicates that this eDCS can be registered multiple times (but is not mandatory)<br>The eDCS from previous observation periods remain unchanged.<br>dt: the eDCS is saved as a time-stream with date-time format.		

For peer review only

The PRISMA model for the AAA bank was constructed with assistance of an experienced information architect of PSI. The eDCSs cover data items ranging from baseline characteristics such as comorbidity to surgical characteristics and postoperative complications. Postoperative complications are registered in codes of the Dutch National Surgical Complications Registry (LHCR), which was established by a committee of the Association of Surgeons of the Netherlands (NVvH).

Data are registered via local data capture platforms, such as Castor EDC[19] (Ciwit, the Netherlands, which is hosted by True[20] in the Netherlands), and are centrally stored in Project Manager Internet Server (ProMISe)[21], a web-based relational database management system (Advanced Data Management, the Netherlands). These systems are compliant with Good Clinical Practice and are ISO27001 certified. All patients are being registered under a study number that is electronically assigned by a designated tool. This study number is used during data collection and data processing.

Biomaterials

Three types of biomaterials are collected: blood, urine and AAA tissue (Table 3).

Table 3. Biomaterials collected for the AAA bank

Biomaterials	Inclusion	Surveillance phase	Surgical phase	Postoperative phase	Storage temperature
Blood - EDTA plasma	once	max. 1 / year	once	Once: 1 year postoperative	≤ -80°C
Blood - Serum	once	max. 1 / year	once	Once: 1 year postoperative	≤ -80°C
Blood - Citrate plasma	once	max. 1 / year	once	Once: 1 year postoperative	≤ -80°C
Blood - EDTA for DNA	once				4°C or ≤ -20°C
Blood - PAXgene for RNA	once				≤ -80°C
Urine	once	max. 1 / year	once	Once: 1 year postoperative	≤ -80°C
Aneurysm tissue - frozen			once		≤ -80°C
Aneurysm tissue – FFPE			once		room temperature

For peer review only - http://bmjopen.bmj.com/site/about/guidelines.xhtml

EDTA = Ethylenediaminetetraacetic acid, FFPE = Formalin-fixed paraffin-embedded

Blood and urine are collected repeatedly during the surveillance phase, up to a maximum of once per year to limit patient burden. Blood is saved as plasma, serum and whole blood for DNA and RNA. When open surgical repair of AAA is performed, aortic tissue is collected, snap frozen and stored at -80°C, and as formalin-fixed paraffin-embedded (FFPE) tissue. After surgery for AAA, blood and urine are collected up to one year postoperatively (Table 3). All procedures concerning biomaterials adhere to standard operating procedures outlined by PSI. Furthermore, all biomaterials are stored in designated PSI biobanks within each UMC. Thus, all biomaterials are stored in the UMC where they are collected.

Imaging data

In order to facilitate future imaging studies, computed tomography (CT) and magnetic resonance imaging (MRI) data that are obtained as part of clinical care are also stored centrally. At the time of writing, the participating centres are harmonizing the CT protocols to acquire standardised images. Partners currently involved in the central storage of images are Translational Research IT (TRaIT, part of Dutch non-profit organisation Lygature) and Vancis, a Dutch service provider of IT services for research with a ISO27001 certificate.[22-24] The imaging data are stored centrally in an Extensible Neuroimaging Archive Toolkit (XNAT) server (Buckner Lab, Harvard University, United States of America).[25] This server is operated by TraIT and is hosted by Vancis.

Imaging data are stored as Digital Imaging and Communications in Medicine (DICOM) data. Before the data are sent to the central server, the study number is allocated to the imaging data to enable linking of imaging data with clinical data and biomaterials. All other identifiable data are removed from the DICOM data using Clinical Trial Processor (CTP, Radiological Society of North America, United States of America).[26]

For peer review only

Statistical analyses

The primary outcomes are AAA growth, AAA rupture and all-cause mortality. AAA growth will be based on multiple measurements and will therefore be analysed with a repeated measures analysis. Potential associations between AAA growth and risk factors that are also measured on multiple time points can be assessed with a multivariate mixed-effects model, while potential associations between AAA rupture and risk factors can be tested with a conditional dynamic prediction model with landmarking.

Due to the expected multifactorial aspect of AAA growth, even weak correlations are of interest to detect. To that end, at least 750 patients are required to be able to detect a correlation coefficient of 0.16 with a power of 80%, and a significance level of .05.

Patient and public involvement

This research was done without patient involvement. Patients were not invited to comment on the study design and were not consulted to develop patient relevant outcomes or interpret the results. Patients were not invited to contribute to the writing or editing of this document for readability or accuracy.

ETHICS AND DISSEMINATION

The Medical Ethics Board and the Biobank Ethics Board of Amsterdam UMC (University of Amsterdam) have approved the AAA bank together with the PARIS study (Table 4) within the scope of the Dutch Medical Research Involving Human Subjects Act (WMO) under registration number NL59991.018.17.

For peer review only

Table 4. The PARIS study, the first proposed study that will be facilitated by the AAA bank

Aims	The aims of the PARIS study are the following: 1) to determine the association between AAA progression (growth or rupture) and the evolution of serum levels of both proteases and cytokines, 2) to determine the association between overall survival and the evolution of serum levels of proteases and cytokines, 3) to determine the association between serum levels of proteases and cytokines and level of proteases and cytokines in AAA tissue, and finally 4) to determine the incidence of, and characterise the type of complications after AAA repair.
Outcomes	The primary endpoints of the PARIS study are: AAA growth, AAA rupture, all-cause mortality, serum levels of cytokines and proteases, and cytokine and protease levels in AAA tissue. Secondary outcomes are the incidence and type of complications after AAA repair.
Sample Size	Because of the multifactorial aspect of AAA progression, we calculated a sample size that will be sufficient for detecting weak correlations between cytokine and protease levels and AAA growth. To detect a correlation coefficient of 0.16 with a power of 80%, and a significance level of .05, a sample size of 750 participants is required. Strategies to achieve sufficient participant enrolment include simple eligibility criteria, and the expressed intention to extend the number of participating centres.
Statistical analysis	The association between AAA growth and the evolution of serum levels of proteases and cytokines will be tested with a multivariate mixed-effects model. For the association between AAA rupture and the evolution of serum levels of proteases and cytokines, a conditional dynamic prediction model with landmarking will be used.

In general, biobanks in the Netherlands do not fall within the scope of the WMO. To be eligible for assessment under WMO, the formulation of a specific research question is required. However, specific research questions are often not present at the moment of biobank initiation.[27] Yet, the submission of the AAA bank together with the PARIS study (that contains a specific research question) enabled approval of the combined project within the scope of the WMO. This approval ensures that the AAA bank adheres to the highest legal and medical-ethical standards, and that participation of other future centres can be realised using the existing procedures of the WMO. Because of this design, all participating patients sign two informed consent forms – one for the biobank and one for the PARIS study (see appendix 1-6 for English versions of the forms). By consenting to the AAA bank and signing its consent form, patients not only consent to the collection and storage of their biomaterials and data,

For peer review only

but also to future analyses of it for research about AAA. Any significant modification to the protocol which may impact patient safety, or the conduct, design or analysis of the study requires formal amendment to the protocol. These will need to be approved by the Medical Ethics Board and the Biobank ethics Board of Amsterdam UMC (University of Amsterdam).

To accommodate patients with different views on data collection, participants can refuse collection of DNA and the sharing of their data with foreign and/or commercial parties. All collected data and biomaterials will be stored for a maximum of 50 years. When a participant decides to withdraw from the AAA bank, all stored biomaterials and data will be destroyed or deleted. When reasonably possible, this is also done with materials that are sent out for a specific study.

Scientific board for future studies

Collected data and biomaterials of the AAA bank can only be used for future studies that fall within the scope of the scientific aims of the AAA bank, and that are approved by the scientific board. Researchers can submit a study proposal with the scientific board of the AAA bank. This board oversees all requests for data and consists of five members, with a minimum of one biostatistician (A.H. Zwinderman, Amsterdam UMC, University of Amsterdam, Department of Clinical Epidemiology) and either a legal expert or ethicist (provided by the String of Pearls Institute) among its members. The other three members are currently vascular surgeons from the initiating UMCs (DAL, WW, JFH). If a study proposal is approved by the scientific board, subsequent medical-ethical approval will be acquired if required by Dutch law or local guidelines. All study proposal require an agreed upon authorship policy prior to submitting a request for data. Furthermore, data is only released in accordance with standard PSI procedures.

Results of individual studies will be published in peer-reviewed scientific journals and will be presented at international conferences.

For peer review only

Acknowledgements

We thank the String of Pearls Institute for their help in initiating and designing the AAA bank and the PARIS study. In addition, we would like to thank Roger Snijder, information architect of PSI, for his help in designing our information model.

For peer review only

References

1. Svensjo S, Bjorck M, Gurtelschmid M, Djavani Gidlund K, Hellberg A, Wanhainen A. Low prevalence of abdominal aortic aneurysm among 65-year-old Swedish men indicates a change in the epidemiology of the disease. Circulation. 2011;124:1118-23.
2. Oliver-Williams C, Sweeting MJ, Turton G, Parkin D, Cooper D, Rodd C, et al. Lessons learned about prevalence and growth rates of abdominal aortic aneurysms from a 25-year ultrasound population screening programme. Br J Surg. 2018;105:68-74.
3. Reimerink JJ, van der Laan MJ, Koelemay MJ, Balm R, Legemate DA. Systematic review and meta-analysis of population-based mortality from ruptured abdominal aortic aneurysm. Br J Surg. 2013;100:1405-13.
4. Nordon IM, Hinchliffe RJ, Loftus IM, Thompson MM. Pathophysiology and epidemiology of abdominal aortic aneurysms. Nat Rev Cardiol. 2011;8:92-102.
5. Brown LC, Powell JT. Risk factors for aneurysm rupture in patients kept under ultrasound surveillance. UK Small Aneurysm Trial Participants. Ann Surg. 1999;230:289-96; discussion 96-7.
6. Hellenthal FA, Buurman WA, Wodzig WK, Schurink GW. Biomarkers of AAA progression. Part 1: extracellular matrix degeneration. Nat Rev Cardiol. 2009;6:464-74.
7. Stather PW, Sidloff DA, Dattani N, Gokani VJ, Choke E, Sayers RD, et al. Meta-analysis and meta-regression analysis of biomarkers for abdominal aortic aneurysm. Br J Surg. 2014;101:1358-72.
8. Deeg MA, Meijer CA, Chan LS, Shen L, Lindeman JH. Prognostic and predictive biomarkers of abdominal aortic aneurysm growth rate. Curr Med Res Opin. 2016;32:509-17.
9. Jalalzadeh H, Indrakusuma R, Planken RN, Legemate DA, Koelemay MJ, Balm R. Inflammation as a Predictor of Abdominal Aortic Aneurysm Growth and Rupture: A Systematic Review of Imaging Biomarkers. Eur J Vasc Endovasc Surg. 2016;52:333-42.
10. Indrakusuma R, Jalalzadeh H, Planken RN, Marquering HA, Legemate DA, Koelemay MJ, et al. Biomechanical Imaging Markers as Predictors of Abdominal Aortic Aneurysm Growth or Rupture: A Systematic Review. Eur J Vasc Endovasc Surg. 2016;52:475-86.
11. Chaikof EL, Dalman RL, Eskandari MK, Jackson BM, Lee WA, Mansour MA, et al. The Society for Vascular Surgery practice guidelines on the care of patients with an abdominal aortic aneurysm. J Vasc Surg. 2018;67:2-77 e2.
12. Manniën J, Ledderhof T, Verspaget HW, Snijder RR, Flikkenschild EF, van Scherrenburg NPC, et al. The Parelsnoer Institute: A National Network of Standardized Clinical Biobanks in the Netherlands. Open Journal of Bioresources. 2017;4.
13. Talmon JL, Ros MG, Legemate DA. PSI: The Dutch Academic Infrastructure for shared biobanks for translational research. Summit Transl Bioinform. 2008;2008:110-4.
14. Nederkoorn PJ, van Dijk EJ, Koudstaal PJ, Luijckx GJ, van Oostenbrugge RJ, Visser MC, et al. The Dutch String-of-Pearls Stroke Study: protocol of a large prospective multicenter genetic cohort study. Int J Stroke. 2015;10:120-2.
15. Aalten P, Ramakers IH, Biessels GJ, de Deyn PP, Koek HL, OldeRikkert MG, et al. The Dutch Parelsnoer Institute--Neurodegenerative diseases; methods, design and baseline results. BMC Neurol. 2014;14:254.
16. Strijker M, Gerritsen A, van Hilst J, Bijlsma MF, Bonsing BA, Brosens LA, et al. The Dutch Pancreas Biobank Within the Parelsnoer Institute: A Nationwide Biobank of Pancreatic and Periampullary Diseases. Pancreas. 2018;47:495-501.
17. van't Riet E, Schram MT, Abbink EJ, Admiraal WM, Dijk-Schaap MW, Holleman F, et al. The Diabetes Pearl: Diabetes biobanking in The Netherlands. BMC Public Health. 2012;12:949.
18. Heelkunde NVv. Richtlijn Diagnostiek en behandeling van het aneurysma van de abdominale aorta. 2009.
19. Castor EDC. www.castoredc.com. Accessed June 1, 2018.
20. True. www.true.nl. Accessed June 1, 2018.
21. Project Manager Internet Server (ProMISe). www.msbi.nl/promise/. Accessed June 1, 2018.

For peer review only

22. Translational Research IT (TraIT). www.ctmm-trait.nl/. Accessed June 1, 2018.
23. Lygature. www.lygature.org/. Accessed June 1, 2018.
24. Vancis. www.vancis.nl/. Accessed June 1, 2018.
25. Marcus DS, Olsen TR, Ramaratnam M, Buckner RL. The Extensible Neuroimaging Archive Toolkit: an informatics platform for managing, exploring, and sharing neuroimaging data. Neuroinformatics. 2007;5:11-34.
26. Radiological Society of North America. www.rsna.org/ctp.aspx. Accessed June 1, 2018.
27. Hendriks AC, van Hellemondt RE. Regulating Privacy and Biobanks in the Netherlands. J Law Med Ethics. 2016;44:68-84.

For peer review only

Authors' contributions

HJ and RI contributed equally to this manuscript. All authors (HJ, RI, JDB, WW, KKY, JHNL, JFH, MJWK, DAL, RB) actively contributed to the study conception, design and its protocol. All authors read and contributed to the writing of the manuscript, and approved the final manuscript.

Funding

The AAA bank is funded by the AMC Foundation, which was not involved in the study design, data collection, or writing of this manuscript. Furthermore, the AMC Foundation will not have any role during its execution, analyses, interpretation of the data, or decision to submit results of this and any future study facilitated by the AAA bank.

Competing interests

The authors declare that they have no competing interests.

Data availability statement

Deidentified clinical data, biomaterials and imaging data are available on reasonable request, as outlined in the main text under 'scientific board for future studies'.

Roles and responsibilities

Principal investigator (RB): final responsibility with regard to design, conduct and protocol (including future revisions) of the AAA bank and the PARIS study. Organises steering committee meetings.

Steering committee (see title page for current members): Agreement of final protocol, recruitment of patients. Reviewing progress of study and if necessary agreeing changes to the protocol to facilitate smooth running of the study.

For peer review only

Lead investigators: in each participating centre a lead investigator (vascular surgeon) has been identified, and is responsible for identification, recruitment, data collection, study follow-up and adherence to the study protocol. Lead investigators are steering committee members.

Trial management committee (principal investigator, research physician and/or data manager): study planning, organising steering committee meetings. Responsible for trial master file.

Since the AAA bank is an observational cohort without any study interventions, a data monitoring committee was not established.

For peer review only

Figures

1. Figure 1. Flow chart of study phases

For peer review only

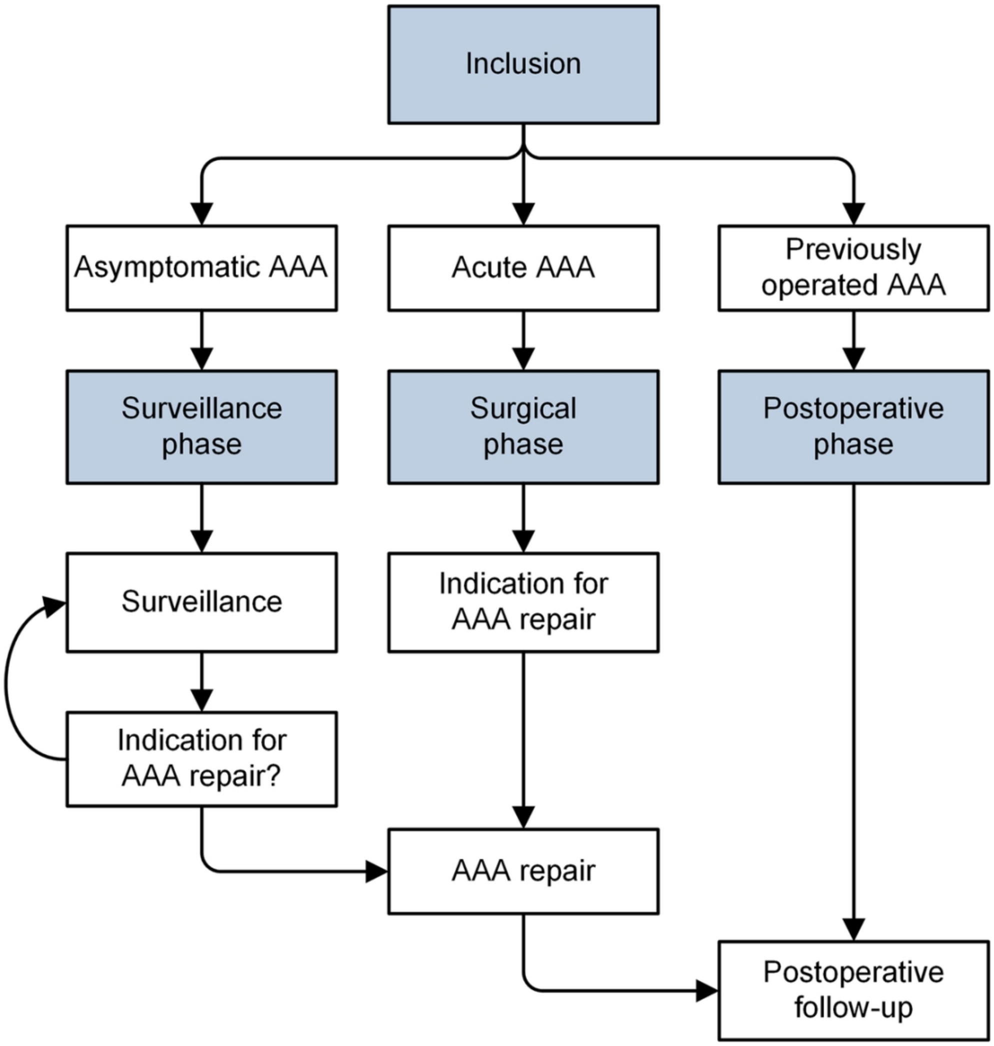

Figure 1. Flow chart of study phases

106x110mm (300 x 300 DPI)

English translation of the informed consent form of the biobank Pearl AAA

Appendix 1. Informed consent form for patients who have an asymptomatic AAA or who have previously undergone AAA repair

Biobank Pearl AAA.

- I consent to the collection, storage and analysis of blood, urine and aortic tissue (in case of open surgery) and data regarding research for abdominal aortic aneurysms.

- I consent to the coded storage of my data for a maximum of 50 years in the biobank Pearl AAA.

- I consent to the coded storage of my biomaterials for a maximum of 50 years for use in future biomedical research.

- I give permission for my GP to be informed that I am participating in this study.

- I give permission for information to be requested from my GP or pharmacy regarding my health status or other (health) registries, under the condition that my privacy will be safeguarded.

- I consent to the possibility that the municipal registry of persons may be consulted so that no errors will be made in contacting study participants.

- In the event of my death, I consent to the possibility that information regarding my death may be acquired from the Central Bureau of Statistics, under the condition that my privacy will be safeguarded.

- During biomedical research with my biomaterials, incidental findings may be found that are associated with a disease or disorder. Sometimes it only concerns an increased risk. I consent that I will be informed about incidental findings if these are relevant to my (or my family's) health status because either prevention or treatment is possible.

- I consent to the possible sharing of my data, biomaterials and imaging data with non-commercial institutions in the Netherlands. This will only be done for research purposes.

- I consent to the possible sharing of my data, biomaterials and imaging data with institutions in a foreign country. This will only be done for research purposes.
    - Yes
    - No

- I consent to the possible sharing of my data, biomaterials and imaging data with commercial entities. This will only be done for research purposes.
    - Yes
    - No

- I consent to the study of my DNA for findings associated with the origin and development of abdominal aortic aneurysms and to the effects and complications of medication
    - Yes
    - No

For peer review only

English translation of the informed consent form of the biobank Pearl AAA

- I consent that I may be approached for additional information and/or biomaterials if this is necessary for a specific biomedical study
  - Yes
  - No

Name participant:
Date of birth:
Signature: Date: __/ __/ __

Declaration of researcher

I hereby declare that I have fully informed this study participant regarding the biobank Pearl AAA. If information comes to light during the course of the study that could affect the study subject's consent, I will inform him/her of this in a timely fashion.

Name researcher:
Signature: Date: __/ __/ __

Additional information has been given by (if applicable):
Name:
Role:
Signature: Date: __/ __/ __

Study participants receive the full information brochure, and a signed copy of the informed consent form.

For peer review only

English translation of the informed consent form of the PARIS study

Appendix 2. Informed consent form for patients who have an asymptomatic AAA or who have previously undergone AAA repair

PARIS study

- I have read the subject information form. I was also able to ask questions. My questions have been answered to my satisfaction. I had enough time to decide whether to participate.
- I know that participation is voluntary. I know that I may decide at any time not to participate after all or to withdraw from the study. I do not need to give a reason for this.
- I give permission for my GP to be informed that I am participating in this study.
- I give permission for information to be requested from my GP or pharmacy regarding my health status.
- I know that some people may have access to all my data to verify the study. These people are listed in this information sheet. I consent to the inspection by them.
- I consent to the collection and use of my data, biomaterials and imaging data as has been specified in this information sheet.
- I consent to the storage of my data for the PARIS study for a maximum of 50 years in the biobank Pearl AAA.
- I consent to the possible sharing of my data, biomaterials and imaging data with non-commercial institutions in the Netherlands. This will only be done for the PARIS study if necessary.
- I consent to the possible sharing of my data, biomaterials and imaging data with institutions in a foreign country. This will only be done for the PARIS study if necessary.
    - Yes
    - No
- I consent to the possible sharing of my data, biomaterials and imaging data with commercial entities. This will only be done for the PARIS study if necessary.
    - Yes
    - No
- I consent to being contacted again after this study for a follow-up study.
    - Yes
    - No
- I want to participate in this study.

Name participant:
Date of birth:
Signature: Date: __/ __/ __

For peer review only

English translation of the informed consent form of the PARIS study

Declaration of researcher

I hereby declare that I have fully informed this study participant regarding the PARIS study. If information comes to light during the course of the study that could affect the study subject's consent, I will inform him/her of this in a timely fashion.

Name researcher:
Signature: Date: __/ __/ __

Additional information has been given by (if applicable):
Name:
Role:
Signature: Date: __/ __/ __

Study participants receive the full information brochure, and a signed copy of the informed consent form.

For peer review only

English translation of the informed consent form of the biobank Pearl AAA

Appendix 3. Informed consent form for patients after emergency AAA repair.

Biobank Pearl AAA

- I consent to the collection, storage and analysis of blood, urine and aortic tissue (in case of open surgery) and data regarding research for abdominal aortic aneurysms.
- I consent to the coded storage of my data for a maximum of 50 years in the biobank Pearl AAA.
- I consent to the coded storage of my biomaterials for a maximum of 50 years for use in future biomedical research.
- I give permission that my GP to be informed that I am participating in this study.
- I give permission for information to be requested from my GP or pharmacy regarding the health status or other (health) registries, under the condition that my privacy will be safeguarded.
- I consent to the possibility that the municipal registry of persons may be consulted so that no errors will be made in contacting study participants.
- In the event of my death, I consent to the possibility that information regarding my death may be acquired from the Central Bureau of Statistics, under the condition that my privacy will be safeguarded.
- During biomedical research with my biomaterials, incidental findings may be found that are associated with a disease or disorder. Sometimes it only concerns an increased risk. I consent that I will be informed about incidental findings if these are relevant to my (or my family's) health status because either prevention or treatment is possible.
- I consent to the possible sharing of my data, biomaterials and imaging data with non-commercial institutions in the Netherlands. This will only be done for research purposes.
- I consent to the possible sharing of my data, biomaterials and imaging data with institutions in a foreign country. This will only be done for research purposes.
    - Yes
    - No
- I consent to the possible sharing of my data, biomaterials and imaging data with commercial entities. This will only be done for research purposes.
    - Yes
    - No
- I consent to the study of my DNA for findings associated with the origin and development of abdominal aortic aneurysms and to the effects and complications of medication
    - Yes
    - No

For peer review only

English translation of the informed consent form of the biobank Pearl AAA

- I consent that I may be approached for additional information and/or biomaterials if this is necessary for a specific biomedical study
  - Yes
  - No

Name participant:
Date of birth:
Signature: Date: __/ __/ __

Declaration of researcher

I hereby declare that I have fully informed the legal representative regarding the biobank Pearl AAA. If information comes to light during the course of the study that could affect the legal representative's consent, I will inform him/her of this in a timely fashion.

Name researcher:
Signature: Date: __/ __/ __

Additional information has been given by (if applicable):
Name:
Role:
Signature: Date: __/ __/ __

Study participants receive the full information brochure, and a signed copy of the informed consent form.

For peer review only

English translation of the informed consent form of the PARIS study

Appendix 4. Informed consent form for patients after emergency AAA repair.

PARIS study

- I have read the subject information form. I was also able to ask questions. My questions have been answered to my satisfaction. I had enough time to decide whether to participate.

- I know that participation is voluntary. I know that I may decide at any time not to participate after all or to withdraw from the study. I do not need to give a reason for this.

- I give permission for my GP to be informed that I am participating in this study.

- I give permission for information to be requested from my GP or pharmacy regarding my health status.

- I know that some people may have access to all my data to verify the study. These people are listed in this information sheet. I consent to the inspection by them.

- I consent to the collection and use of my data, biomaterials and imaging data as has been specified in this information sheet.

- I consent to the storage of my data for the PARIS study for a maximum of 50 years in the biobank Pearl AAA.

- I consent to the possible sharing of my data, biomaterials and imaging data with non-commercial institutions in the Netherlands. This will only be done for the PARIS study if necessary.

- I consent to the possible sharing of my data, biomaterials and imaging data with institutions in a foreign country. This will only be done for the PARIS study if necessary.
    - Yes
    - No

- I consent to the possible sharing of my data, biomaterials and imaging data with commercial entities. This will only be done for the PARIS study if necessary.
    - Yes
    - No

- I consent to being contacted again after this study for a follow-up study.
    - Yes
    - No

- I want to participate in this study.

Name participant:
Date of birth:
Signature: Date: __/ __/ __

Declaration of researcher

For peer review only

English translation of the informed consent form of the PARIS study

I hereby declare that I have fully informed this study participant regarding the PARIS study. If information comes to light during the course of the study that could affect the study subject's consent, I will inform him/her of this in a timely fashion.

Name researcher:
Signature: Date: __/ __/ __

Additional information has been given by (if applicable):
Name:
Role:
Signature: Date: __/ __/ __

Study participants receive the full information brochure, and a signed copy of the informed consent form.

For peer review only

English translation of the informed consent form of the biobank Pearl AAA

Appendix 5. Informed consent form for legal representative.

Biobank Pearl AAA

I have been asked to give written consent for the participation of the following person in the biobank Parel AAA.

Name person:
Date of birth: __/ __/ __

- I consent to the collection, storage and analysis of blood, urine and aortic tissue (in case of open surgery) and data of the person regarding research for abdominal aortic aneurysms.

- I consent to the coded storage of the data for a maximum of 50 years in the biobank Pearl AAA.

- I consent to the coded storage of the biomaterials for a maximum of 50 years for use in future biomedical research.

- I give permission that the GP of the person is to be informed that the person is participating in this study.

- I give permission for information regarding the person to be requested from the GP or pharmacy regarding the health status or other (health) registries, under the condition that his/her privacy will be safeguarded.

- During biomedical research with the biomaterials, incidental findings may be found that are associated with a disease or disorder. Sometimes it only concerns an increased risk. I consent that the GP of the person will be informed about incidental findings if these are relevant to the family's health status because either prevention or treatment is possible.

- I consent to the possible sharing of the data, biomaterials and imaging data with non-commercial institutions in the Netherlands. This will only be done for research purposes.

- I consent to the possible sharing of the data, biomaterials and imaging data with institutions in a foreign country. This will only be done for research purposes.
    - Yes
    - No

- I consent to the possible sharing of the data, biomaterials and imaging data with commercial entities. This will only be done for research purposes.
    - Yes
    - No

- I consent to the study of the DNA for findings associated with the origin and development of abdominal aortic aneurysms and to the effects and complications of medication
    - Yes
    - No

For peer review only

English translation of the informed consent form of the biobank Pearl AAA

Name legal representative:
Date of birth:
Relation to the person:
Signature: Date: __/ __/ __

Declaration of researcher

I hereby declare that I have fully informed the legal representative regarding the biobank Pearl AAA. If information comes to light during the course of the study that could affect the legal representative's consent, I will inform him/her of this in a timely fashion.

Name researcher:
Signature: Date: __/ __/ __

Additional information has been given by (if applicable):
Name:
Role:
Signature: Date: __/ __/ __

Study participants receive the full information brochure, and a signed copy of the informed consent form.

For peer review only

English translation of the informed consent form of the PARIS study

Appendix 6. Informed consent form for legal representative.

PARIS study

I have been asked to give written consent for the participation of the following person in the PARIS study.

Name person:
Date of birth: __/ __/ __

- I have read the subject information form. I was also able to ask questions. My questions have been answered to my satisfaction. I had enough time to decide whether this person participates.

- I know that participation is voluntary. I know that I may decide at any time that this person does not to participate after all or to withdraw him/her from the study. I do not need to give a reason for this.

- I give permission for the GP of this person to be informed that he/she is participating in this study.

- I give permission for information to be requested from the GP or pharmacy of this person regarding his/her health status.

- I know that some people may have access to all this person's data to verify the study. These people are listed in this information sheet. I consent to the inspection by them.

- I consent to the collection and use of the data, biomaterials and imaging data as has been specified in this information sheet.

- I consent to the storage of the data for the PARIS study for a maximum of 50 years in the biobank Pearl AAA.

- I consent to the possible sharing of the data, biomaterials and imaging data with non-commercial institutions in the Netherlands. This will only be for the PARIS study if necessary.

- I consent to the possible sharing of the data, biomaterials and imaging data with institutions in a foreign country. This will only be done for the PARIS study.
    - Yes
    - No

- I consent to the possible sharing of the data, biomaterials and imaging data with commercial entities. This will only be done for the PARIS study.
    - Yes
    - No

- I agree with the participation of this person with the study.

For peer review only

English translation of the informed consent form of the PARIS study

Name legal representative:
Date of birth:
Relation to the person:
Signature: Date: __/ __/ __

Declaration of researcher

I hereby declare that I have fully informed the legal representative regarding the biobank Pearl AAA. If information comes to light during the course of the study that could affect the legal representative's consent, I will inform him/her of this in a timely fashion.

Name researcher:
Signature: Date: __/ __/ __

Additional information has been given by (if applicable):
Name:
Role:
Signature: Date: __/ __/ __

Study participants receive the full information brochure, and a signed copy of the informed consent form.

For peer review only

SPIRIT

STANDARD PROTOCOL ITEMS: RECOMMENDATIONS FOR INTERVENTIONAL TRIALS

SPIRIT 2013 Checklist: Recommended items to address in a clinical trial protocol and related documents*

Section/item	Item No	Description	Page, paragraph
**Administrative information**			
Title	1	Descriptive title identifying the study design, population, interventions, and, if applicable, trial acronym	p.1
Trial registration	2a	Trial identifier and registry name. If not yet registered, name of intended registry	p.4: trial registration p.8: study design
	2b	All items from the World Health Organization Trial Registration Data Set	p.8: table 1
Protocol version	3	Date and version identifier	p.9: study design
Funding	4	Sources and types of financial, material, and other support	p.22: funding
Roles and responsibilitie s	5a	Names, affiliations, and roles of protocol contributors	p.1,2 p.22: authors' contributions
	5b	Name and contact information for the trial sponsor	p.2, correspondin g author is sponsor-investigator p.8: table 1
	5c	Role of study sponsor and funders, if any, in study design; collection, management, analysis, and interpretation of data; writing of the report; and the decision to submit the report for publication, including whether they will have ultimate authority over any of these activities	p.22: funding
	5d	Composition, roles, and responsibilities of the coordinating centre, steering committee, endpoint adjudication committee, data management team, and other individuals or groups overseeing the trial, if applicable (see Item 21a for data monitoring committee)	p.22: roles and responsibiliti es

For peer review only

**Introduction**			
Background and rationale	6a	Description of research question and justification for undertaking the trial, including summary of relevant studies (published and unpublished) examining benefits and harms for each intervention	p.7: final paragraph
	6b	Explanation for choice of comparators	NA
Objectives	7	Specific objectives or hypotheses	p.7: final paragraph p.17: table 4
Trial design	8	Description of trial design including type of trial (eg, parallel group, crossover, factorial, single group), allocation ratio, and framework (eg, superiority, equivalence, noninferiority, exploratory)	p.8: study design
**Methods: Participants, interventions, and outcomes**			
Study setting	9	Description of study settings (eg, community clinic, academic hospital) and list of countries where data will be collected. Reference to where list of study sites can be obtained	p.8-9: study design
Eligibility criteria	10	Inclusion and exclusion criteria for participants. If applicable, eligibility criteria for study centres and individuals who will perform the interventions (eg, surgeons, psychotherapists)	p.10: study population
Interventions	11a	Interventions for each group with sufficient detail to allow replication, including how and when they will be administered	NA
	11b	Criteria for discontinuing or modifying allocated interventions for a given trial participant (eg, drug dose change in response to harms, participant request, or improving/worsening disease)	NA
	11c	Strategies to improve adherence to intervention protocols, and any procedures for monitoring adherence (eg, drug tablet return, laboratory tests)	NA
	11d	Relevant concomitant care and interventions that are permitted or prohibited during the trial	NA
Outcomes	12	Primary, secondary, and other outcomes, including the specific measurement variable (eg, systolic blood pressure), analysis metric (eg, change from baseline, final value, time to event), method of aggregation (eg, median, proportion), and time point for each outcome. Explanation of the clinical relevance of chosen efficacy and harm outcomes is strongly recommended	p.8: table 1 p.17: table 4
Participant timeline	13	Time schedule of enrolment, interventions (including any run-ins and washouts), assessments, and visits for participants. A schematic diagram is highly recommended (see Figure)	p.10-11: study procedures Fig 1.

For peer review only - http://bmjopen.bmj.com/site/about/guidelines.xhtml

Sample size	14	Estimated number of participants needed to achieve study objectives and how it was determined, including clinical and statistical assumptions supporting any sample size calculations	p.17: table 4
Recruitment	15	Strategies for achieving adequate participant enrolment to reach target sample size	p.10: study procedures p.17: table 4

Methods: Assignment of interventions (for controlled trials)

Allocation:

Sequence generation	16a	Method of generating the allocation sequence (eg, computer-generated random numbers), and list of any factors for stratification. To reduce predictability of a random sequence, details of any planned restriction (eg, blocking) should be provided in a separate document that is unavailable to those who enrol participants or assign interventions	NA
Allocation concealme nt mechanis m	16b	Mechanism of implementing the allocation sequence (eg, central telephone; sequentially numbered, opaque, sealed envelopes), describing any steps to conceal the sequence until interventions are assigned	NA
Implement ation	16c	Who will generate the allocation sequence, who will enrol participants, and who will assign participants to interventions	NA
Blinding (masking)	17a	Who will be blinded after assignment to interventions (eg, trial participants, care providers, outcome assessors, data analysts), and how	NA
	17b	If blinded, circumstances under which unblinding is permissible, and procedure for revealing a participant's allocated intervention during the trial	NA

Methods: Data collection, management, and analysis

Data collection methods	18a	Plans for assessment and collection of outcome, baseline, and other trial data, including any related processes to promote data quality (eg, duplicate measurements, training of assessors) and a description of study instruments (eg, questionnaires, laboratory tests) along with their reliability and validity, if known. Reference to where data collection forms can be found, if not in the protocol	p.11: clinical data – PRISMA p.14: biomaterials
	18b	Plans to promote participant retention and complete follow-up, including list of any outcome data to be collected for participants who discontinue or deviate from intervention protocols	p.10: study procedures p.14: biomaterials

For peer review only - http://bmjopen.bmj.com/site/about/guidelines.xhtml

Data management	19	Plans for data entry, coding, security, and storage, including any related processes to promote data quality (eg, double data entry; range checks for data values). Reference to where details of data management procedures can be found, if not in the protocol	p.11: clinical data - PRISMA
Statistical methods	20a	Statistical methods for analysing primary and secondary outcomes. Reference to where other details of the statistical analysis plan can be found, if not in the protocol	p.17: table 4
	20b	Methods for any additional analyses (eg, subgroup and adjusted analyses)	NA
	20c	Definition of analysis population relating to protocol non-adherence (eg, as randomised analysis), and any statistical methods to handle missing data (eg, multiple imputation)	NA
**Methods: Monitoring**			
Data monitoring	21a	Composition of data monitoring committee (DMC); summary of its role and reporting structure; statement of whether it is independent from the sponsor and competing interests; and reference to where further details about its charter can be found, if not in the protocol. Alternatively, an explanation of why a DMC is not needed	p.23 final sentence
	21b	Description of any interim analyses and stopping guidelines, including who will have access to these interim results and make the final decision to terminate the trial	NA
Harms	22	Plans for collecting, assessing, reporting, and managing solicited and spontaneously reported adverse events and other unintended effects of trial interventions or trial conduct	NA
Auditing	23	Frequency and procedures for auditing trial conduct, if any, and whether the process will be independent from investigators and the sponsor	NA
**Ethics and dissemination**			
Research ethics approval	24	Plans for seeking research ethics committee/institutional review board (REC/IRB) approval	p.17 p.18: Scientific board for future studies
Protocol amendments	25	Plans for communicating important protocol modifications (eg, changes to eligibility criteria, outcomes, analyses) to relevant parties (eg, investigators, REC/IRBs, trial participants, trial registries, journals, regulators)	p.18

For peer review only - http://bmjopen.bmj.com/site/about/guidelines.xhtml

Consent or assent	26a	Who will obtain informed consent or assent from potential trial participants or authorised surrogates, and how (see Item 32)	p.10: recruitment procedure
	26b	Additional consent provisions for collection and use of participant data and biological specimens in ancillary studies, if applicable	appendix 1-6
Confidentiality	27	How personal information about potential and enrolled participants will be collected, shared, and maintained in order to protect confidentiality before, during, and after the trial	p.14: clinical data - PRISMA
Declaration of interests	28	Financial and other competing interests for principal investigators for the overall trial and each study site	p.22: competing interests
Access to data	29	Statement of who will have access to the final trial dataset, and disclosure of contractual agreements that limit such access for investigators	p.18: scientific board<br>p.9: table 1 IPD sharing statement
Ancillary and post-trial care	30	Provisions, if any, for ancillary and post-trial care, and for compensation to those who suffer harm from trial participation	NA
Dissemination policy	31a	Plans for investigators and sponsor to communicate trial results to participants, healthcare professionals, the public, and other relevant groups (eg, via publication, reporting in results databases, or other data sharing arrangements), including any publication restrictions	p.18: scientific board
	31b	Authorship eligibility guidelines and any intended use of professional writers	p.18: scientific board
	31c	Plans, if any, for granting public access to the full protocol, participant-level dataset, and statistical code	p.18: scientific board<br>p.9: table 1 IPD sharing statement
**Appendices**			
Informed consent materials	32	Model consent form and other related documentation given to participants and authorised surrogates	appendix 1-6

For peer review only - http://bmjopen.bmj.com/site/about/guidelines.xhtml

Biological specimens	33	Plans for collection, laboratory evaluation, and storage of biological specimens for genetic or molecular analysis in the current trial and for future use in ancillary studies, if applicable	p.14: biomaterials

*It is strongly recommended that this checklist be read in conjunction with the SPIRIT 2013 Explanation & Elaboration for important clarification on the items. Amendments to the protocol should be tracked and dated. The SPIRIT checklist is copyrighted by the SPIRIT Group under the Creative Commons "Attribution-NonCommercial-NoDerivs 3.0 Unported" license.

For peer review only

BMJ Open

Design and protocol of a comprehensive multicentre biobank for abdominal aortic aneurysms

Journal:	*BMJ Open*
Manuscript ID	bmjopen-2018-028858.R1
Article Type:	Protocol
Date Submitted by the Author:	21-Mar-2019
Complete List of Authors:	Jalalzadeh, Hamid; Amsterdam UMC, Surgery<br>Indrakusuma, Reza; Amsterdam UMC, Surgery<br>Blankensteijn, Jan; Amsterdam UMC, Surgery<br>Wisselink, Willem; Amsterdam UMC, Surgery<br>Yeung, Kakkhee; Amsterdam UMC, Surgery<br>Lindeman, Jan; Leiden University Medical Center, Vascular surgery<br>Hamming, Jaap; LUMC, surgery<br>Koelemay, Mark; Amsterdam UMC, Surgery<br>Legemate, Dink; Amsterdam UMC, Surgery<br>Balm, Ron; Amsterdam UMC, Surgery
**Primary Subject Heading**:	Surgery
Secondary Subject Heading:	Cardiovascular medicine
Keywords:	Vascular surgery < SURGERY, VASCULAR SURGERY, Cardiovascular imaging < RADIOLOGY & IMAGING

SCHOLARONE™
Manuscripts

Design and protocol of a comprehensive multicentre biobank for abdominal aortic aneurysms

Hamid Jalalzadeh*[1], Reza Indrakusuma*[1], Jan D. Blankensteijn[2], Willem Wisselink[2], Kak K. Yeung[2], Jan H.N. Lindeman[3], Jaap F. Hamming[3], Mark J.W. Koelemay[1], Dink A. Legemate[1], Ron Balm[1]

* Both authors contributed equally

1. Amsterdam UMC, University of Amsterdam, Department of Surgery, Amsterdam Cardiovascular Sciences, Meibergdreef 9, 1105 AZ, Amsterdam, The Netherlands
2. Amsterdam UMC, Vrije Universiteit Amsterdam, Department of Surgery, Amsterdam Cardiovascular Sciences, De Boelelaan 1117, 1081 HV, Amsterdam, The Netherlands
3. Leiden University Medical Center, Department of Surgery, Albinusdreef 2, 2333 ZA, Leiden, The Netherlands

Current steering committee:

Hamid Jalalzadeh: h.jalalzadeh@amc.uva.nl

Reza Indrakusuma: r.indrakusuma@amc.uva.nl

Jan D. Blankensteijn: j.blankensteijn@vumc.nl

Willem Wisselink: w.wisselink@vumc.nl

Kak. K. Yeung: k.yeung@vumc.nl

Jan H.N. Lindeman: j.h.n.lindeman@lumc.nl

Jaap F. Hamming: j.f.hamming@lumc.nl

Mark. J.W. Koelemay: m.j.koelemaij@amc.uva.nl

Dink A. Legemate: d.a.legemate@amc.uva.nl

Ron Balm: r.balm@amc.nl

For peer review only

Corresponding author and sponsor-investigator:

Ron Balm

r.balm@amc.nl

telephone number: +31205667832

Amsterdam UMC, University of Amsterdam, Department of Surgery, Amsterdam Cardiovascular

Sciences, Meibergdreef 9, 1105 AZ, Amsterdam, The Netherlands

Licence per instructions

I, the Submitting Author has the right to grant and does grant on behalf of all authors of the Work (as defined in the below author licence), an exclusive licence and/or a non-exclusive licence for contributions from authors who are: i) UK Crown employees; ii) where BMJ has agreed a CC-BY licence shall apply, and/or iii) in accordance with the terms applicable for US Federal Government officers or employees acting as part of their official duties; on a worldwide, perpetual, irrevocable, royalty-free basis to BMJ Publishing Group Ltd ("BMJ") its licensees and where the relevant Journal is co-owned by BMJ to the co-owners of the Journal, to publish the Work in BMJ Open and any other BMJ products and to exploit all rights, as set out in our licence.

The Submitting Author accepts and understands that any supply made under these terms is made by BMJ to the Submitting Author unless you are acting as an employee on behalf of your employer or a postgraduate student of an affiliated institution which is paying any applicable article publishing charge ("APC") for Open Access articles. Where the Submitting Author wishes to make the Work available on an Open Access basis (and intends to pay the relevant APC), the terms of reuse of such Open Access shall be governed by a Creative Commons licence – details of these licences and which Creative Commons licence will apply to this Work are set out in our licence referred to above.

Word count:

- Abstract: 324
- Manuscript excluding title page, abstract, tables, acknowledgements, contributions and references: 2674

For peer review only

ABSTRACT

Introduction

The pathophysiology and natural course of abdominal aortic aneurysms (AAAs) are insufficiently understood. In order to improve our understanding, it is imperative to carry out longitudinal research which combines biomarkers with clinical and imaging data measured over multiple time points. Therefore, a multicentre biobank, databank and imagebank has been established in the Netherlands: the '*Pearl Abdominal Aortic Aneurysm*' (AAA bank).

Methods and analysis

The AAA bank is a prospective multicentre observational bio-, data- and imagebank of patients with an AAA. It is embedded within the framework of the String of Pearls Institute (PSI) which facilitates uniform biobanking in all university medical centres (UMCs) in the Netherlands. The AAA bank has been initiated by the two UMCs of Amsterdam UMC and by Leiden University Medical Center. Participants will be followed during AAA follow-up. Clinical data are collected every patient contact. Three types of biomaterials are collected at baseline and during follow-up: blood (including DNA and RNA), urine and AAA tissue if open surgical repair is performed. Imaging data that are obtained as part of clinical care are stored in the imagebank. All data and biomaterials are processed and stored in a standardised manner. AAA growth will be based on multiple measurements and will be analysed with a repeated measures analysis. Potential associations between AAA growth and risk factors that are also measured on multiple time points can be assessed with multivariable mixed-effects models, while potential associations between AAA rupture and risk factors can be tested with a conditional dynamic prediction model with landmarking, or with joint models in which linear mixed-effects models are combined with Cox regression.

Ethics and dissemination

The AAA bank is approved by the Medical ethics Board of the Amsterdam UMC (University of Amsterdam).

For peer review only

Registration: this study was retrospectively registered on October 25, 2017 on www.clinicaltrials.gov (NCT03320408).

Keywords:

Abdominal aortic aneurysm, biobank, databank, imagebank, pathophysiology, natural history

For peer review only

Strengths and limitations of this study

- Longitudinal collection of clinical data, biomaterials and imaging data of patients with an abdominal aortic aneurysm provides ample opportunity to better understand the natural history, and to search for prognostic risk factors that might benefit future treatment.
- The inclusion of patients with a small asymptomatic abdominal aortic aneurysm offers the possibility to study natural history from an early stage.
- Standardised collection and storage of biomaterials allows the analysis of patients included at different hospitals.
- Study follow-up is done at routine follow-up appointments to minimise participant burden, yet also means that study follow-up will vary between patients.
- Patient recruitment currently only takes place in university medical centres which are tertiary referral centres.

For peer review only

INTRODUCTION

An abdominal aortic aneurysm (AAA) is a focal dilatation of the abdominal aorta that affects mostly elderly men. The prevalence of AAA in the general population is 1.3-2.2% in 65-year old men.[1, 2] An AAA is an asymptomatic disorder which is associated with a high risk of mortality in case of rupture.[3] Yet, the risk of rupture itself is difficult to measure accurately and also varies considerably between patients. Consequently, the management of patients with an asymptomatic AAA is focused on balancing the risk of rupture with other competing risks of death, with the aim of preserving quality and quantity of life. On the one hand, asymptomatic patients who are estimated to have a high risk of rupture, in general at a diameter of more than 5.5 cm for men, may be offered prophylactic AAA repair if the risk of rupture outweighs any procedural and/or competing risks.[4] On the other hand, asymptomatic patients for whom the risk of rupture is estimated to be smaller than procedural and/or competing risks will be offered surveillance – for example those with an AAA diameter smaller than 5.5 cm or those with severe comorbidities. A large body of research has been dedicated to determining the optimal surgical treatment for AAA, focusing on either the threshold diameter for repair, the method of repair, or the outcome and follow-up after treatment.

Although many studies have tried to unravel AAA pathophysiology, this aspect is still insufficiently understood. Most of the current knowledge originates from histopathological studies that reflect the end stage of AAA disease. Early determinants and drivers of AAA formation are largely unidentified due to the unavailability of AAA tissue from an early stage of the disease. Therefore, the known determinants of AAA development are limited to general risk factors such as male sex, ageing, smoking or connective tissue diseases.[5, 6]

Recent studies focused on biomarker research to find early disease markers and potential targets for pharmacological treatment. Promising circulating biomarkers included markers of matrix turnover (matrix metalloproteinases), markers of inflammation (interleukins and C-reactive protein) and markers of lipid metabolism (lipoproteins).[7-9] Unfortunately, to date, none of these biomarkers have found their way to clinical practice. This is mostly due to their low prognostic value for AAA

For peer review only

progression, and because many studies have not corrected for factors such as smoking or comorbidities.[8] The same applies to new imaging biomarkers such as ^{18}F-fluoro-deoxy-glucose (^{18}F-FDG), or biomechanical markers such as peak wall stress and wall shear stress.[10-12]

To better understand AAA pathophysiology and its natural course, it is imperative to combine studies with biomarkers, imaging markers, and longitudinal data. To that end, a multicentre databank, biobank and imagebank has been established in the Netherlands: the '*Pearl Abdominal Aortic Aneurysm*, hereafter referred to as the AAA bank. The aim of this project is to facilitate future studies on AAA. It is part of the Dutch String of Pearls Institute (PSI), which facilitates uniform biobanking in all eight university medical centres (UMCs) in the Netherlands.[13] The systematic collection of clinical data, biomaterials and imaging data will enable a diverse range of studies on patients with AAA. The AAA bank will especially focus on patients with a small AAA to collect longitudinal data early on in the development of AAA.

The first proposed study that will be carried out with the collected biomaterials is the 'Predicting aneurysm growth and rupture with longitudinal biomarkers' (PARIS) study. The PARIS study aims to determine the association between AAA progression and the evolution of serum levels of proteases and cytokines.

The scientific aims of the AAA bank are (1) to gain insight in the pathophysiology and natural history of AAA; (2) to gain more knowledge about the rupture risk of AAA; and (3) to evaluate and improve treatment of patients with an AAA. Future studies with data from the AAA bank must adhere to these scientific aims.

For peer review only

METHODS AND ANALYSIS

Study design

The AAA bank is a prospective multicentre observational biobank, databank and imagebank of patients with AAA in The Netherlands (ClinicalTrials.gov: NCT03320408, see table 1 for the WHO Trial Registration Data Set).

Table 1. WHO trial registration data set

Primary registry and trial identifying number	ClinicalTrials.gov: NCT03320408
Date of registration in primary registry	25 October 2017
Secondary identifying numbers	NL59991.018.17, PARIS study, biobank Pearl AAA
Sources of monetary of material support	AMC Foundation for monetary support.
Primary sponsor	Academic Medical Center – University of Amsterdam
Secondary sponsor(s)	none
Contact for public queries	Els Kuiters, e.kuiters@amc.uva.nl, +31205667832, Meibergdreef 9, 1105AZ, Amsterdam, the Netherlands.
Contact for scientific queries	Principal investigator: prof. R. Balm, r.balm@amc.nl, +3120-5667832, Meibergdreef 9, 1105AZ, Amsterdam, The Netherlands.<br><br>Els Kuiters, e.kuiters@amc.uva.nl, +31205667832, Meibergdreef 9, 1105AZ, Amsterdam, the Netherlands.
Public title	PARIS study & biobank Pearl AAA
Scientific title	Predicting aneurysm growth and rupture with longitudinal biomarkers (PARIS study) & biobank Pearl AAA
Countries of recruitment	The Netherlands
Health condition(s)	Abdominal aortic aneurysm
Intervention(s)	None
Key inclusion and exclusion criteria	Inclusion criteria: adult with an AAA, or who has been previously treated for an AAA. Adequate comprehension of the Dutch language to provide written informed consent.<br>Exclusion criteria: Decisionally impaired patients. The exception are patients who are decisionally impaired due to the effects of an acute AAA for whom a separate recruitment and consent procedure exists.
Study type	Observational longitudinal patient registry and biobank
Date of first enrolment	4 October 2017
Sample size	Planned: 750<br>Currently enrolled: 87
Recruitment status	Recruiting; participants are currently being recruited and enrolled
Primary outcome(s)	Outcome: AAA growth. Time frame: up to 10 years of follow-up<br>Outcome. AAA rupture. Time frame: up to 10 years of follow-up<br>Outcome: all-cause mortality. Time frame: up to 10 years of follow-up<br>Outcome: evolution of serum levels of proteases and cytokines. Time frame:

For peer review only

	a maximum of 1 measurement annually up to 10 years of follow-up<br>Outcome: proteases and cytokine levels in AAA tissue. Time frame: if open AAA repair is performed and AAA tissue is collected. This is a one-time measurement.
Key secondary outcome(s)	Outcome: incidence and type of complications after AAA repair. Time frame: up to 10 years of follow-up after AAA repair
Ethics review	Status: approved<br>Date of approval: 25 August 2017<br>Name and contact details of ethics committees:<br>Medical Ethics Board of Amsterdam UMC (University of Amsterdam). mecamc@amc.uva.nl, +31205667389, Trinity building C, fourth floor, Pietersbergweg 17, 1105BM Amsterdam, the Netherlands.<br><br>Biobank Ethics Board of Amsterdam UMC (University of Amsterdam), biobanktoetsing@amc.uva.nl, +31205666730, Meibergdreef 9, 1105AZ, Amsterdam, the Netherlands.
Completion date	Expected: 4 October 2032
Summary results	No results yet.
IPD sharing statement	Plan to share IPD: Yes<br>Plan description: IPD sharing for research will be allowed in a data sharing procedure. Scientific requests need to fall under the scope of the scientific aims as formulated in this manuscript. Researchers willing to requests IPD can initiate this procedure by contacting the researchers. Data will be released depending on the scientific quality of the submitted request.

AAA = abdominal aortic aneurysm

The active protocol at the time of writing is version 3, 22 December 2017. The AAA bank is embedded within the framework of PSI, which is co-financed by the Dutch government and the Netherlands Federation of University Medical centres (NFU).[13] PSI was established in 2007 and aims to facilitate standardised nationwide biobanks and clinical databases.[14] At the time of writing, 17 different patient cohorts (*Pearls*) for different diseases have been initiated within the PSI framework. Examples include the Diabetes Pearl, the Pearl Neurodegenerative Diseases, the Stroke Pearl, and the Dutch Pancreas biobank.[15-18] All these biobanks adhere to an internal regulatory framework, which prescribes legal and ethical rules concerning the conduct of all Pearl-related activities.[14]

The AAA bank has been initiated by the two UMCs from Amsterdam University Medical Centers (University of Amsterdam and Vrije Universiteit Amsterdam) and by Leiden University Medical Center (LUMC). In accordance with the goals of PSI and NFU, the AAA bank aims to expand to the other Dutch UMCs. The AAA bank is currently an ongoing project, with active recruitment and collection of data and biomaterials. The first patient was recruited on October 4, 2017.

For peer review only

Study population

All capable adults with AAA in participating university medical centres are eligible for inclusion in the AAA bank. This also includes patients who previously have undergone AAA repair. Patients who are incapable due to a ruptured AAA (RAAA) can also be included, using a special recruitment process, which will be described below. All included patients will be followed for as long as they visit their treating vascular surgeon.

Recruitment procedure

Eligible patients are recruited at the inpatient or outpatient clinic of the department of vascular surgery of the participating hospitals by research physicians and/or data managers. Participants who agree to participate give written informed consent during their visit to the inpatient or outpatient clinic. When patients arrive in an emergency setting with a ruptured or symptomatic AAA, oral consent is required from either the patient or a legal representative. This oral consent has to be confirmed in writing at a later stage – either by the patient, or by a representative in case of a fatal outcome. In the event that no written informed consent can be obtained, all data and biomaterials collected for the AAA bank will be destroyed.

Study procedures

The study procedures of the AAA bank are embedded within regular AAA treatment and are carried out by physician-researchers and data managers. Standard operating procedures have been set up to minimise the amount of missing data. Clinical data and biomaterials are collected during and after visits that are already part of clinical treatment, to limit participant burden and improve participant retention. No research visits will be planned for the AAA bank. In addition, only imaging data that is obtained as part of clinical care will be stored in the AAA bank.

For peer review only

In line with regular AAA management, four distinct study phases have been identified: inclusion phase, surveillance phase, surgical phase, and postoperative phase (Figure 1; blue boxes). Within each phase, multiple visits can take place – especially in the surveillance or postoperative phase. These phases can theoretically continue indefinitely, depending on the course of the AAA. With regard to the 'surveillance phase', the Dutch AAA guideline advises that patients visit a vascular surgeon at intervals of two years, one year, or three months, depending on the AAA diameter.[19] Patients can move from the 'surveillance phase' to the 'surgical phase' and subsequently to the 'postoperative phase' (Figure 1). Furthermore, there will be patients who at some point reach the threshold diameter for repair, yet who do not undergo repair for various reasons such as severe comorbidity. In these cases, an individual clinical decision – unrelated to their participation with the AAA bank – will have to be made together with the patient whether surveillance continues with regular intervals or whether the patient chooses to quit with surveillance altogether. Patients who choose to continue with surveillance will still be asked for biomaterials and clinical data, while patients who quit surveillance will be considered lost to follow-up in future analyses.

Clinical data – PRISMA

All clinical data are collected according to an information model called Parelsnoer Repository for Information Specification, Modelling and Architecture (PRISMA), which was constructed with the assistance of an experienced information architect of PSI. The majority of clinical data will be collected from electronic health records (EHRs) in order to reduce participant burden, while only a minority of the data will be collected through questionnaires, as outlined in table 2.

For peer review only

Table 2. The PRISMA information model of the AAA bank

<table>
<tr><th>eDCS name</th><th colspan="3">Inclusion</th><th colspan="3">Follow-up[N]</th><th>Stream</th><th>Source</th></tr>
<tr><th></th><th>Asymptomatic AAA</th><th>Operation for acute AAA</th><th>Previously operated AAA</th><th>Surveillance phase</th><th>Surgical phase</th><th>Postoperative phase</th><th></th><th></th></tr>
<tr><td>Patient information</td><td colspan="3">1</td><td colspan="3">M</td><td></td><td>EHR</td></tr>
<tr><td>Informed consent</td><td colspan="3">1</td><td colspan="3">M</td><td></td><td>n.a.</td></tr>
<tr><td>Living situation</td><td colspan="3">1</td><td colspan="3">1</td><td></td><td>Q</td></tr>
<tr><td>Current aneurysm state</td><td colspan="3">1</td><td colspan="3">1</td><td></td><td>EHR</td></tr>
<tr><td>First occurrence comorbidity</td><td colspan="3">1</td><td colspan="3">M</td><td></td><td>EHR</td></tr>
<tr><td>Family history</td><td colspan="3">0..n</td><td colspan="3">M</td><td></td><td>Q</td></tr>
<tr><td>Social history</td><td colspan="3">1</td><td colspan="3">M</td><td></td><td>Q</td></tr>
<tr><td>Initial aneurysm characteristics</td><td colspan="3">1</td><td colspan="3"></td><td></td><td>EHR</td></tr>
<tr><td>Comorbidity status</td><td colspan="3">1</td><td colspan="3">1</td><td></td><td>EHR</td></tr>
<tr><td>Medication</td><td colspan="3">1</td><td colspan="3">1</td><td></td><td>EHR, Q</td></tr>
<tr><td>Intoxications</td><td colspan="3">1</td><td colspan="3">1</td><td></td><td>EHR, Q</td></tr>
<tr><td>Physical examination</td><td colspan="3">1</td><td>1</td><td>1</td><td></td><td></td><td>EHR, Q</td></tr>
<tr><td>Blood test results</td><td colspan="3">1</td><td colspan="3">1</td><td></td><td>EHR</td></tr>
<tr><td>Surveillance</td><td>1</td><td colspan="2"></td><td>1</td><td colspan="2"></td><td></td><td>EHR</td></tr>
<tr><td>Preoperative assessment</td><td></td><td colspan="2">1</td><td></td><td>1</td><td></td><td></td><td>EHR</td></tr>
<tr><td>AAA repair</td><td></td><td colspan="2">1</td><td></td><td>1</td><td></td><td></td><td>EHR</td></tr>
<tr><td>Postoperative admission</td><td></td><td colspan="2">1</td><td></td><td>1</td><td></td><td></td><td>EHR</td></tr>
<tr><td>Postoperative follow-up</td><td></td><td colspan="2">1</td><td></td><td></td><td>1</td><td></td><td>EHR</td></tr>
</table>

For peer review only

Biomaterials	1	1		n.a.
Cardiovascular events			dt	EHR
Other surgical procedures			dt	EHR
Malignancies			dt	EHR
AAA imaging	0..n		dt	EHR
Complications			dt	EHR
Imaging data	1	1	dt	EHR
	eDCS: electronic Data Capture System;<br>1: indicates that an eDCS is registered once and is overwritten in case of future changes.<br>M: Means 'Modify', and indicates that the eDCS can be modified within the same observation period. N: Indicates that this eDCS can be registered multiple times (but at least once)<br>0..n: Indicates that this eDCS can be registered multiple times (but is not mandatory)<br>The eDCS from previous observation periods remain unchanged.<br>dt: the eDCS is saved as a time-stream with date-time format.<br>EHR: electronic health records<br>Q: questionnaire			

For peer review only

Furthermore, PRISMA consists of electronic Data Capture Systems (eDCSs), with each eDCS covering a certain theme, as described in more detail in Table 3.

Table 3. General description of each eDCS in PRISMA

eDCS	General description
Patient information	Study number, year of birth and survival status including cause of death.
Informed consent	Status of informed consent (given or withdrawn), date of informed consent, individual patient decisions with regard to additional consent options.
Living situation	e.g. independent or assisted living.
Current aneurysm state	e.g. asymptomatic, acute or postoperative status.
First occurrence comorbidity	This eDCS contains a predefined list of comorbidities. Only if a participant has any of these comorbidities will this be registered, including the initial date of diagnosis.<br><br>The list includes:<br>Hypertension, COPD, idiopathic lung fibrosis, peptic ulcer, diabetes mellitus, hypo- and hyperthyroidism, hepatitis, portal hypertension, liver cirrhosis, any other liver disease, peripheral arterial disease, intracranial aneurysm, popliteal aneurysm, any other aneurysm, connective

For peer review only

	tissue disease, hypercholesterolaemia, carotid artery disease, renal dysfunction, renal disorders, ischaemic heart disease, heart failure, heart arrhythmia, heart valve disorders, any other cardiac disorder, hemi- or paraplegia, dementia and AIDS.<br><br>Free text descriptions can be provided for cases with an “any other” comorbidity as mentioned above.
Family history	Registration of each known family member with (a history of) aortic aneurysmal disease and if known, at what age and whether there was an aortic rupture.
Social history	Marital status, employment status, educational status.
Initial aneurysm characteristics	Pathogenesis and type of AAA, status of AAA at time of inclusion, date of initial diagnosis, shape of AAA.
Comorbidity status	Status of a predefined list of comorbidities. This will be registered if this is known at the time of a visit to the department of vascular surgery.<br><br>The list includes:<br>COPD, diabetes mellitus (e.g. dependency on

For peer review only

	insulin), peripheral arterial disease (e.g. Fontaine classification), renal dysfunction, angina pectoris, heart failure.
Medication	This eDCS contains a predefined list of medications. Only pills and injections will be registered. If a medication is not included in the list, it will be added via a free text description. Dosage will be registered for cardiovascular medications.<br><br>The list includes:<br>All types of cardiovascular medications, diabetes mellitus medication, corticosteroids, immunosuppressive medication and benzodiazepines.
Intoxications	Nicotine and alcohol use.
Physical examination	Body weight and patient length.
Blood test results	This eDCS contains a predefined list of blood tests, which will be registered if these have been carried out for clinical care.<br><br>The list includes:<br>Creatinine, estimated glomerular filtration rate, total cholesterol, high-density lipoprotein cholesterol, low-density lipoprotein cholesterol,

For peer review only

	triglycerides,
Surveillance	Date of surveillance visit, treatment plan including motivation.
Preoperative assessment	American Society of Anaesthesiologists physical status classification
AAA repair	Date of AAA repair, type of AAA repair, intraoperative complications
Postoperative admission	Length of hospitalisation, intensive care hospitalisation
Postoperative follow-up	Date of postoperative follow-up visit, treatment plan including motivation
Cardiovascular events	Myocardial infarction, cerebrovascular accident, thromboembolic events including dates.
Other surgical procedures	This eDCS contains a predefined list of certain procedures.<br><br>The list includes:<br>Cardiac surgery, vascular surgery, abdominal and thoracic surgery, and transplant surgery.
Malignancies	General type of malignancy, date of diagnosis, type of treatments.
AAA imaging	Date and type of AAA imaging. AAA status at time of imaging (e.g. asymptomatic, acute or postoperative), AAA diameter,
Complications	Complications after any AAA repair will be

For peer review only

	registered according to a predefined code list of the Dutch National Surgical Complications Registry (LHCR), which was established by a committee of the Association of Surgeons in the Netherlands (NVvH).

Data are registered via local data capture platforms, such as Castor EDC[20] (Ciwit, the Netherlands, which is hosted by True[21] in the Netherlands), and are centrally stored in Project Manager Internet Server (ProMISe)[22], a web-based relational database management system (Advanced Data Management, the Netherlands). These systems are compliant with Good Clinical Practice and are ISO27001 certified. All patients are being registered under a study number that is electronically assigned by a designated tool. This study number is used during data collection and data processing.

Biomaterials

Three types of biomaterials are collected: blood, urine and AAA tissue (Table 4).

Table 4. Biomaterials collected for the AAA bank

Biomaterials	Inclusion	Surveillance phase	Surgical phase	Postoperative phase	Storage temperature
Blood - EDTA plasma	once	max. 1 / year	once	Once: 1 year postoperative	≤ -80°C
Blood - Serum	once	max. 1 / year	once	Once: 1 year postoperative	≤ -80°C
Blood - Citrate plasma	once	max. 1 / year	once	Once: 1 year postoperative	≤ -80°C
Blood - EDTA for DNA	once				4°C or ≤ -20°C
Blood - PAXgene for RNA	once				≤ -80°C
Urine	once	max. 1 / year	once	Once: 1 year postoperative	≤ -80°C
Aneurysm tissue - frozen			once		≤ -80°C
Aneurysm tissue – FFPE			once		room temperature

EDTA = Ethylenediaminetetraacetic acid, FFPE = Formalin-fixed paraffin-embedded

For peer review only

Blood and urine are collected repeatedly during the surveillance phase, up to a maximum of once per year to limit patient burden. Blood is saved as plasma, serum and whole blood for DNA and RNA. When open surgical repair of AAA is performed, aortic tissue is collected, snap frozen and stored at -80°C, and as formalin-fixed paraffin-embedded (FFPE) tissue. After surgery for AAA, blood and urine are collected up to one year postoperatively (Table 4). All procedures concerning biomaterials adhere to standard operating procedures outlined by PSI. Furthermore, all biomaterials are stored in designated PSI biobanks within each UMC. Thus, all biomaterials are stored in the UMC where they are collected.

Imaging data

In order to facilitate future imaging studies, computed tomography (CT) and magnetic resonance imaging (MRI) data that are obtained as part of clinical care are also stored centrally. At the time of writing, the participating centres are harmonizing the CT protocols to acquire standardised images. Partners currently involved in the central storage of images are Translational Research IT (TRaIT, part of Dutch non-profit organisation Lygature) and Vancis, a Dutch service provider of IT services for research with a ISO27001 certificate.[23-25] The imaging data are stored centrally in an Extensible Neuroimaging Archive Toolkit (XNAT) server (Buckner Lab, Harvard University, United States of America).[26] This server is operated by TraIT and is hosted by Vancis.

Imaging data are stored as Digital Imaging and Communications in Medicine (DICOM) data. Before the data are sent to the central server, the study number is allocated to the imaging data to enable linking of imaging data with clinical data and biomaterials. All other identifiable data are removed from the DICOM data using Clinical Trial Processor (CTP, Radiological Society of North America, United States of America).[27]

Statistical analyses

For peer review only

The primary outcomes are AAA growth, AAA rupture and all-cause mortality. AAA growth will be based on multiple measurements and will therefore be analysed with a repeated measures analysis. Potential associations between AAA growth and risk factors that are also measured on multiple time points can be assessed with multivariable mixed-effects models, while potential associations between AAA rupture and risk factors can be tested with a conditional dynamic prediction model with landmarking, or with joint models in which linear mixed-effects models are combined with Cox regression.

Due to the expected multifactorial aspect of AAA growth, even weak correlations are of interest to detect. To that end, at least 750 patients are required to be able to detect a correlation coefficient of 0.16 with a power of 80%, and a significance level of .05.

Patient and public involvement

This research was done without patient involvement. Patients were not invited to comment on the study design and were not consulted to develop patient relevant outcomes or interpret the results. Patients were not invited to contribute to the writing or editing of this document for readability or accuracy.

ETHICS AND DISSEMINATION

The Medical Ethics Board and the Biobank Ethics Board of Amsterdam UMC (University of Amsterdam) have approved the AAA bank together with the PARIS study (Table 5) within the scope of the Dutch Medical Research Involving Human Subjects Act (WMO) under registration number NL59991.018.17.

For peer review only

Table 5. The PARIS study, the first proposed study that will be facilitated by the AAA bank

Aims	The aims of the PARIS study are: 1) to determine the association between AAA progression (growth or rupture) and the evolution of serum levels of both proteases and cytokines, 2) to determine the association between overall survival and the evolution of serum levels of proteases and cytokines, 3) to determine the association between serum levels of proteases and cytokines and level of proteases and cytokines in AAA tissue, and finally 4) to determine the incidence of, and characterise the type of complications after AAA repair.
Outcomes	The primary outcomes are: AAA growth, AAA rupture, all-cause mortality, serum levels of cytokines and proteases, and cytokine and protease levels in AAA tissue. Secondary outcomes are the incidence and type of complications after AAA repair.
Sample Size	Because of the multifactorial aspect of AAA progression, we calculated a sample size that will be sufficient for detecting weak correlations between cytokine and protease levels and AAA growth. To detect a correlation coefficient of 0.16 with a power of 80%, and a significance level of .05, a sample size of 750 participants is required. Strategies to achieve sufficient participant enrolment include simple eligibility criteria, and the expressed intention to extend the number of participating centres.
Statistical analysis	Categorical variables will be presented as numbers and percentages, and will be compared between groups with the Chi-square test of Fisher exact test where appropriate. Continuous variables will be presented as means ± standard deviation or as medians with the interquartile range, depending on distribution. Distribution of continuous variables will be tested with the Shapiro-Wilk test. Continuous variables will be compared between groups with either the unpaired t-test or the Mann–Whitney *U* test depending on distribution.<br><br>Individual AAA growth (based on repeated AAA diameter measurements) and the evolution of serum levels of cytokines and proteases will each be analysed with linear mixed-effects models to estimate the slope of temporal change (i.e. the evolution through time). The linear mixed-effects model that estimates the slope for the evolution of each biomarker will also adjust for potentially relevant covariables including but not limited to sex, age and cardiovascular comorbidity. A forward stepwise selection will be used to select only those covariables that were significant in univariable analysis. Finally, correlations coefficient will be estimated between the slope of AAA progression and the slope of each biomarker.<br><br>Freedom from AAA rupture and all-cause mortality will be estimated with the Kaplan-Meier method, while joint modelling will be used to assess the association between the evolution of serum levels of proteases and cytokines and AAA rupture and all-cause mortality. A joint model will be performed per biomarker. In order to assess the association, joint modelling combines linear mixed-effects models for the estimation of slope of temporal change per biomarker with Cox regression for the analysis of freedom

For peer review only

	from AAA rupture and all-cause mortality, in order to estimate hazard ratios. The estimated slope and value of the studied biomarker will be added as covariables in each joint model. Furthermore, both models will be adjusted for potentially relevant covariables, including but not limited to sex, age, most recent AAA diameter and cardiovascular comorbidity using the same selection procedure. In addition, AAA growth instead of the most recent AAA diameter will be analysed in a separate multivariable joint model. In all analyses a p value $< .05$ will be considered statistically significant, including the covariable selection procedure.

In general, biobanks in the Netherlands do not fall within the scope of the WMO. To be eligible for assessment under WMO, the formulation of a specific research question is required. However, specific research questions are often not present at the moment of biobank initiation.[28] Yet, the submission of the AAA bank together with the PARIS study (that contains a specific research question) enabled approval of the combined project within the scope of the WMO. This approval ensures that the AAA bank adheres to the highest legal and medical-ethical standards, and that participation of other future centres can be realised using the existing procedures of the WMO. Because of this design, all participating patients sign two informed consent forms – one for the biobank and one for the PARIS study (see appendix 1-6 for English versions of the forms). By consenting to the AAA bank and signing its consent form, patients not only consent to the collection and storage of their biomaterials and data, but also to future analyses of it for research about AAA. Any significant modification to the protocol which may impact patient safety, or the conduct, design or analysis of the study requires formal amendment to the protocol. These will need to be approved by the Medical Ethics Board and the Biobank ethics Board of Amsterdam UMC (University of Amsterdam).

To accommodate patients with different views on data collection, participants can refuse collection of DNA and the sharing of their data with foreign and/or commercial parties. All collected data and biomaterials will be stored for a maximum of 50 years. When a participant decides to withdraw from the AAA bank, all stored biomaterials and data will be destroyed or deleted. When reasonably possible, this is also done with materials that are sent out for a specific study.

For peer review only

Scientific board for future studies

Collected data and biomaterials of the AAA bank can only be used for future studies that fall within the scope of the scientific aims of the AAA bank, and that are approved by the scientific board. Researchers can submit a study proposal with the scientific board of the AAA bank. Each study proposal must include a study objective and/or research questions, the type of data and biomaterials required, a statistical analysis plan and an agreed upon authorship policy. This board oversees all requests for data and consists of five members, with a minimum of one biostatistician (A.H. Zwinderman, Amsterdam UMC, University of Amsterdam, Department of Clinical Epidemiology) and either a legal expert or ethicist (provided by the String of Pearls Institute) among its members. The other three members are currently vascular surgeons from the initiating UMCs (DAL, WW, JFH). If a study proposal is approved by the scientific board, subsequent medical-ethical approval will be acquired if required by Dutch law or local guidelines. Furthermore, data is only released in accordance with standard PSI procedures.

Results of individual studies will be published in peer-reviewed scientific journals and will be presented at international conferences.

For peer review only

Acknowledgements

We thank the String of Pearls Institute for their help in initiating and designing the AAA bank and the PARIS study. In addition, we would like to thank Roger Snijder, information architect of PSI, for his help in designing our information model.

For peer review only

References

1. Svensjo S, Bjorck M, Gurtelschmid M, Djavani Gidlund K, Hellberg A, Wanhainen A. Low prevalence of abdominal aortic aneurysm among 65-year-old Swedish men indicates a change in the epidemiology of the disease. Circulation. 2011;124:1118-23.
2. Oliver-Williams C, Sweeting MJ, Turton G, Parkin D, Cooper D, Rodd C, et al. Lessons learned about prevalence and growth rates of abdominal aortic aneurysms from a 25-year ultrasound population screening programme. Br J Surg. 2018;105:68-74.
3. Reimerink JJ, van der Laan MJ, Koelemay MJ, Balm R, Legemate DA. Systematic review and meta-analysis of population-based mortality from ruptured abdominal aortic aneurysm. Br J Surg. 2013;100:1405-13.
4. Wanhainen A, Verzini F, Van Herzeele I, Allaire E, Bown M, Cohnert T, et al. Editor's Choice - European Society for Vascular Surgery (ESVS) 2019 Clinical Practice Guidelines on the Management of Abdominal Aorto-iliac Artery Aneurysms. Eur J Vasc Endovasc Surg. 2019;57:8-93.
5. Nordon IM, Hinchliffe RJ, Loftus IM, Thompson MM. Pathophysiology and epidemiology of abdominal aortic aneurysms. Nat Rev Cardiol. 2011;8:92-102.
6. Brown LC, Powell JT. Risk factors for aneurysm rupture in patients kept under ultrasound surveillance. UK Small Aneurysm Trial Participants. Ann Surg. 1999;230:289-96; discussion 96-7.
7. Hellenthal FA, Buurman WA, Wodzig WK, Schurink GW. Biomarkers of AAA progression. Part 1: extracellular matrix degeneration. Nat Rev Cardiol. 2009;6:464-74.
8. Stather PW, Sidloff DA, Dattani N, Gokani VJ, Choke E, Sayers RD, et al. Meta-analysis and meta-regression analysis of biomarkers for abdominal aortic aneurysm. Br J Surg. 2014;101:1358-72.
9. Deeg MA, Meijer CA, Chan LS, Shen L, Lindeman JH. Prognostic and predictive biomarkers of abdominal aortic aneurysm growth rate. Curr Med Res Opin. 2016;32:509-17.
10. Jalalzadeh H, Indrakusuma R, Planken RN, Legemate DA, Koelemay MJ, Balm R. Inflammation as a Predictor of Abdominal Aortic Aneurysm Growth and Rupture: A Systematic Review of Imaging Biomarkers. Eur J Vasc Endovasc Surg. 2016;52:333-42.
11. Indrakusuma R, Jalalzadeh H, Planken RN, Marquering HA, Legemate DA, Koelemay MJ, et al. Biomechanical Imaging Markers as Predictors of Abdominal Aortic Aneurysm Growth or Rupture: A Systematic Review. Eur J Vasc Endovasc Surg. 2016;52:475-86.
12. Chaikof EL, Dalman RL, Eskandari MK, Jackson BM, Lee WA, Mansour MA, et al. The Society for Vascular Surgery practice guidelines on the care of patients with an abdominal aortic aneurysm. J Vasc Surg. 2018;67:2-77 e2.
13. Manniën J, Ledderhof T, Verspaget HW, Snijder RR, Flikkenschild EF, van Scherrenburg NPC, et al. The Parelsnoer Institute: A National Network of Standardized Clinical Biobanks in the Netherlands. Open Journal of Bioresources. 2017;4.
14. Talmon JL, Ros MG, Legemate DA. PSI: The Dutch Academic Infrastructure for shared biobanks for translational research. Summit Transl Bioinform. 2008;2008:110-4.
15. Nederkoorn PJ, van Dijk EJ, Koudstaal PJ, Luijckx GJ, van Oostenbrugge RJ, Visser MC, et al. The Dutch String-of-Pearls Stroke Study: protocol of a large prospective multicenter genetic cohort study. Int J Stroke. 2015;10:120-2.
16. Aalten P, Ramakers IH, Biessels GJ, de Deyn PP, Koek HL, OldeRikkert MG, et al. The Dutch Parelsnoer Institute--Neurodegenerative diseases; methods, design and baseline results. BMC Neurol. 2014;14:254.
17. Strijker M, Gerritsen A, van Hilst J, Bijlsma MF, Bonsing BA, Brosens LA, et al. The Dutch Pancreas Biobank Within the Parelsnoer Institute: A Nationwide Biobank of Pancreatic and Periampullary Diseases. Pancreas. 2018;47:495-501.
18. van't Riet E, Schram MT, Abbink EJ, Admiraal WM, Dijk-Schaap MW, Holleman F, et al. The Diabetes Pearl: Diabetes biobanking in The Netherlands. BMC Public Health. 2012;12:949.
19. Heelkunde NVv. Richtlijn Diagnostiek en behandeling van het aneurysma van de abdominale aorta. 2009.

For peer review only

20. Castor EDC. www.castoredc.com. Accessed June 1, 2018.
21. True. www.true.nl. Accessed June 1, 2018.
22. Project Manager Internet Server (ProMISe). www.msbi.nl/promise/. Accessed June 1, 2018.
23. Translational Research IT (TraIT). www.ctmm-trait.nl/. Accessed June 1, 2018.
24. Lygature. www.lygature.org/. Accessed June 1, 2018.
25. Vancis. www.vancis.nl/. Accessed June 1, 2018.
26. Marcus DS, Olsen TR, Ramaratnam M, Buckner RL. The Extensible Neuroimaging Archive Toolkit: an informatics platform for managing, exploring, and sharing neuroimaging data. Neuroinformatics. 2007;5:11-34.
27. Radiological Society of North America. www.rsna.org/ctp.aspx. Accessed June 1, 2018.
28. Hendriks AC, van Hellemondt RE. Regulating Privacy and Biobanks in the Netherlands. J Law Med Ethics. 2016;44:68-84.

For peer review only

Authors' contributions

HJ and RI contributed equally to this manuscript. All authors (HJ, RI, JDB, WW, KKY, JHNL, JFH, MJWK, DAL, RB) actively contributed to the study conception, design and its protocol. All authors read and contributed to the writing of the manuscript, and approved the final manuscript.

Funding

The AAA bank is funded by the AMC Foundation, which was not involved in the study design, data collection, or writing of this manuscript. Furthermore, the AMC Foundation will not have any role during its execution, analyses, interpretation of the data, or decision to submit results of this and any future study facilitated by the AAA bank.

Competing interests

The authors declare that they have no competing interests.

Data availability statement

Deidentified clinical data, biomaterials and imaging data are available on reasonable request, as outlined in the main text under ‘scientific board for future studies’.

Roles and responsibilities

Principal investigator (RB): final responsibility with regard to design, conduct and protocol (including future revisions) of the AAA bank and the PARIS study. Organises steering committee meetings.

Steering committee (see title page for current members): Agreement of final protocol, recruitment of patients. Reviewing progress of study and if necessary agreeing changes to the protocol to facilitate smooth running of the study.

For peer review only

Lead investigators: in each participating centre a lead investigator (vascular surgeon) has been identified, and is responsible for identification, recruitment, data collection, study follow-up and adherence to the study protocol. Lead investigators are steering committee members.

Trial management committee (principal investigator, research physician and/or data manager): study planning, organising steering committee meetings. Responsible for trial master file.

Since the AAA bank is an observational cohort without any study interventions, a data monitoring committee was not established.

For peer review only

Figures

1. Figure 1. Flow chart of study phases

For peer review only

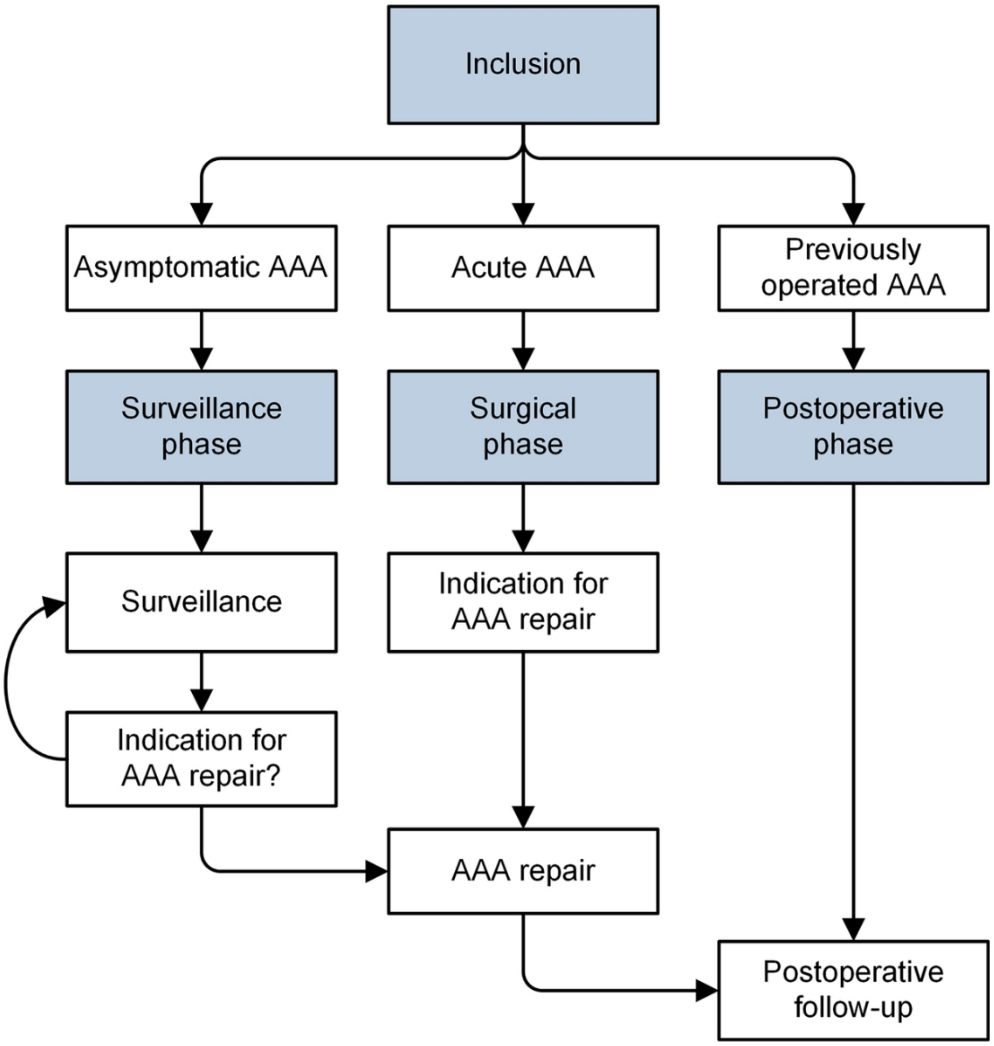

Figure 1. Flow chart of study phases

106x110mm (300 x 300 DPI)

English translation of the informed consent form of the biobank Pearl AAA

Appendix 1. Informed consent form for patients who have an asymptomatic AAA or who have previously undergone AAA repair

Biobank Pearl AAA.

- I consent to the collection, storage and analysis of blood, urine and aortic tissue (in case of open surgery) and data regarding research for abdominal aortic aneurysms.
- I consent to the coded storage of my data for a maximum of 50 years in the biobank Pearl AAA.
- I consent to the coded storage of my biomaterials for a maximum of 50 years for use in future biomedical research.
- I give permission for my GP to be informed that I am participating in this study.
- I give permission for information regarding my health status to be requested from my GP, pharmacy and the municipal registry of persons, under the condition that my privacy will be safeguarded.
- During biomedical research with my biomaterials, incidental findings may be found that are associated with a disease or disorder. Sometimes it only concerns an increased risk. I consent that I will be informed about incidental findings if these are relevant to my (or my family's) health status because either prevention or treatment is possible.
- I consent to the possible sharing of my data, biomaterials and imaging data with non-commercial institutions in countries in the European Union. This will only be done for research purposes.

Additional consent options:

- I consent to the acquisition of my data from national registries concerning the quality of healthcare and its improvement, under the condition that my privacy will be safeguarded.
    - Yes
    - No
- In the event of my death, I consent to the possibility that information regarding my death may be acquired from the Central Bureau of Statistics, under the condition that my privacy will be safeguarded.
    - Yes
    - No
- I consent to the possible sharing of my data, biomaterials and imaging data with institutions in countries outside the European Union. This will only be done for research purposes.
    - Yes
    - No
- I consent to the possible sharing of my data, biomaterials and imaging data with commercial entities. This will only be done for research purposes.
    - Yes

For peer review only

English translation of the informed consent form of the biobank Pearl AAA

    - No

- I consent to the study of my DNA for findings associated with the origin and development of abdominal aortic aneurysms and to the effects and complications of medication
    - Yes
    - No

- I consent that I may be approached for additional information and/or biomaterials if this is necessary for a specific biomedical study
    - Yes
    - No

Name participant:
Date of birth:
Signature: Date: __/ __/ __

Declaration of researcher

I hereby declare that I have fully informed this study participant regarding the biobank Pearl AAA. If information comes to light during the course of the study that could affect the study subject's consent, I will inform him/her of this in a timely fashion.

Name researcher:
Signature: Date: __/ __/ __

Additional information has been given by (if applicable):
Name:
Role:
Signature: Date: __/ __/ __

Study participants receive the full information brochure, and a signed copy of the informed consent form.

For peer review only

English translation of the informed consent form of the PARIS study

Appendix 2. Informed consent form for patients who have an asymptomatic AAA or who have previously undergone AAA repair

PARIS study

- I have read the subject information form. I was also able to ask questions. My questions have been answered to my satisfaction. I had enough time to decide whether to participate.
- I know that participation is voluntary. I know that I may decide at any time not to participate after all or to withdraw from the study. I do not need to give a reason for this.
- I give permission for my GP to be informed that I am participating in this study.
- I give permission for information to be requested from my GP or pharmacy regarding my health status.
- I know that some people may have access to all my data to verify the study. These people are listed in this information sheet. I consent to the inspection by them.
- I consent to the collection and use of my data, biomaterials and imaging data as has been specified in this information sheet.
- I consent to the storage of my data for the PARIS study for a maximum of 50 years in the biobank Pearl AAA.
- I consent to the possible sharing of my data, biomaterials and imaging data with non-commercial institutions in countries in the European Union. This will only be done for the PARIS study if necessary.

Additional consent options:

- I consent to the possible sharing of my data, biomaterials and imaging data with institutions in countries outside the European Union. This will only be done for the PARIS study if necessary.
    - Yes
    - No
- I consent to the possible sharing of my data, biomaterials and imaging data with commercial entities. This will only be done for the PARIS study if necessary.
    - Yes
    - No
- I consent to being contacted again after this study for a follow-up study.
    - Yes
    - No
- I want to participate in this study.

For peer review only

English translation of the informed consent form of the PARIS study

Name participant:
Date of birth:
Signature: Date: __/ __/ __

Declaration of researcher

I hereby declare that I have fully informed this study participant regarding the PARIS study. If information comes to light during the course of the study that could affect the study subject's consent, I will inform him/her of this in a timely fashion.

Name researcher:
Signature: Date: __/ __/ __

Additional information has been given by (if applicable):
Name:
Role:
Signature: Date: __/ __/ __

Study participants receive the full information brochure, and a signed copy of the informed consent form.

For peer review only

English translation of the informed consent form of the biobank Pearl AAA

Appendix 3. Informed consent form for patients after emergency AAA repair.

Biobank Pearl AAA

- I consent to the collection, storage and analysis of blood, urine and aortic tissue (in case of open surgery) and data regarding research for abdominal aortic aneurysms.
- I consent to the coded storage of my data for a maximum of 50 years in the biobank Pearl AAA.
- I consent to the coded storage of my biomaterials for a maximum of 50 years for use in future biomedical research.
- I give permission that my GP to be informed that I am participating in this study.
- I give permission for information regarding my health status to be requested from my GP, pharmacy and the municipal registry of persons, under the condition that my privacy will be safeguarded.
- During biomedical research with my biomaterials, incidental findings may be found that are associated with a disease or disorder. Sometimes it only concerns an increased risk. I consent that I will be informed about incidental findings if these are relevant to my (or my family's) health status because either prevention or treatment is possible.
- I consent to the possible sharing of my data, biomaterials and imaging data with non-commercial institutions in countries in the European Union. This will only be done for research purposes.

- I consent to the acquisition of my data from national registries concerning the quality of healthcare and its improvement, under the condition that my privacy will be safeguarded.
    - Yes
    - No
- In the event of my death, I consent to the possibility that information regarding my death may be acquired from the Central Bureau of Statistics, under the condition that my privacy will be safeguarded.
    - Yes
    - No
- I consent to the possible sharing of my data, biomaterials and imaging data with institutions in countries outside the European Union. This will only be done for research purposes.
    - Yes
    - No
- I consent to the possible sharing of my data, biomaterials and imaging data with commercial entities. This will only be done for research purposes.
    - Yes
    - No

For peer review only

English translation of the informed consent form of the biobank Pearl AAA

- I consent to the study of my DNA for findings associated with the origin and development of abdominal aortic aneurysms and to the effects and complications of medication
    - Yes
    - No

- I consent that I may be approached for additional information and/or biomaterials if this is necessary for a specific biomedical study
    - Yes
    - No

Name participant:
Date of birth:
Signature: Date: __/ __/ __

Declaration of researcher

I hereby declare that I have fully informed the legal representative regarding the biobank Pearl AAA. If information comes to light during the course of the study that could affect the legal representative's consent, I will inform him/her of this in a timely fashion.

Name researcher:
Signature: Date: __/ __/ __

Additional information has been given by (if applicable):
Name:
Role:
Signature: Date: __/ __/ __

Study participants receive the full information brochure, and a signed copy of the informed consent form.

For peer review only

English translation of the informed consent form of the PARIS study

Appendix 4. Informed consent form for patients after emergency AAA repair.

PARIS study

- I have read the subject information form. I was also able to ask questions. My questions have been answered to my satisfaction. I had enough time to decide whether to participate.
- I know that participation is voluntary. I know that I may decide at any time not to participate after all or to withdraw from the study. I do not need to give a reason for this.
- I give permission for my GP to be informed that I am participating in this study.
- I give permission for information to be requested from my GP or pharmacy regarding my health status.
- I know that some people may have access to all my data to verify the study. These people are listed in this information sheet. I consent to the inspection by them.
- I consent to the collection and use of my data, biomaterials and imaging data as has been specified in this information sheet.
- I consent to the storage of my data for the PARIS study for a maximum of 50 years in the biobank Pearl AAA.
- I consent to the possible sharing of my data, biomaterials and imaging data with non-commercial institutions in countries in the European Union. This will only be done for the PARIS study if necessary.

Additional consent options:

- I consent to the possible sharing of my data, biomaterials and imaging data with institutions in countries outside the European Union. This will only be done for the PARIS study if necessary.
  - Yes
  - No
- I consent to the possible sharing of my data, biomaterials and imaging data with commercial entities. This will only be done for the PARIS study if necessary.
  - Yes
  - No
- I consent to being contacted again after this study for a follow-up study.
  - Yes
  - No
- I want to participate in this study.

Name participant:
Date of birth:

For peer review only

English translation of the informed consent form of the PARIS study

Signature: Date: __/ __/ __

Declaration of researcher

I hereby declare that I have fully informed this study participant regarding the PARIS study. If information comes to light during the course of the study that could affect the study subject's consent, I will inform him/her of this in a timely fashion.

Name researcher:
Signature: Date: __/ __/ __

Additional information has been given by (if applicable):
Name:
Role:
Signature: Date: __/ __/ __

Study participants receive the full information brochure, and a signed copy of the informed consent form.

For peer review only

English translation of the informed consent form of the biobank Pearl AAA

Appendix 5. Informed consent form for legal representative.

Biobank Pearl AAA

I have been asked to give written consent for the participation of the following person in the biobank Parel AAA.

Name person:
Date of birth: __/ __/ __

- I consent to the collection, storage and analysis of blood, urine and aortic tissue (in case of open surgery) and data of the person regarding research for abdominal aortic aneurysms.
- I consent to the coded storage of the data for a maximum of 50 years in the biobank Pearl AAA.
- I consent to the coded storage of the biomaterials for a maximum of 50 years for use in future biomedical research.
- I give permission that the GP of the person is to be informed that the person is participating in this study.
- I give permission for information regarding the health status of the person to be requested from the GP and pharmacy, under the condition that his/her privacy will be safeguarded.
- During biomedical research with the biomaterials, incidental findings may be found that are associated with a disease or disorder. Sometimes it only concerns an increased risk. I consent that the GP of the person will be informed about incidental findings if these are relevant to the family's health status because either prevention or treatment is possible.
- I consent to the possible sharing of the data, biomaterials and imaging data with non-commercial institutions in countries the European Union. This will only be done for research purposes.

Additional consent options:

- I consent to the acquisition of data regarding the person from national registries concerning the quality of healthcare and its improvement, under the condition that his/her privacy will be safeguarded.
    - Yes
    - No
- I consent to the possible sharing of the data, biomaterials and imaging data of the person with institutions in countries outside the European Union. This will only be done for research purposes.
    - Yes
    - No
- I consent to the possible sharing of the data, biomaterials and imaging data with commercial entities. This will only be done for research purposes.

For peer review only

English translation of the informed consent form of the biobank Pearl AAA

- Yes
- No

- I consent to the study of the DNA for findings associated with the origin and development of abdominal aortic aneurysms and to the effects and complications of medication
  - Yes
  - No

Name legal representative:
Date of birth:
Relation to the person:
Signature: Date: __/ __/ __

Declaration of researcher

I hereby declare that I have fully informed the legal representative regarding the biobank Pearl AAA. If information comes to light during the course of the study that could affect the legal representative's consent, I will inform him/her of this in a timely fashion.

Name researcher:
Signature: Date: __/ __/ __

Additional information has been given by (if applicable):
Name:
Role:
Signature: Date: __/ __/ __

Study participants receive the full information brochure, and a signed copy of the informed consent form.

For peer review only

English translation of the informed consent form of the PARIS study

Appendix 6. Informed consent form for legal representative.

PARIS study

I have been asked to give written consent for the participation of the following person in the PARIS study.

Name person:
Date of birth: __/ __/ __

- I have read the subject information form. I was also able to ask questions. My questions have been answered to my satisfaction. I had enough time to decide whether this person participates.
- I know that participation is voluntary. I know that I may decide at any time that this person does not to participate after all or to withdraw him/her from the study. I do not need to give a reason for this.
- I give permission for the GP of this person to be informed that he/she is participating in this study.
- I give permission for information to be requested from the GP or pharmacy of this person regarding his/her health status.
- I know that some people may have access to all this person's data to verify the study. These people are listed in this information sheet. I consent to the inspection by them.
- I consent to the collection and use of the data, biomaterials and imaging data as has been specified in this information sheet.
- I consent to the storage of the data for the PARIS study for a maximum of 50 years in the biobank Pearl AAA.
- I consent to the possible sharing of the data, biomaterials and imaging data with non-commercial institutions in countries in the European Union. This will only be for the PARIS study if necessary.

- I consent to the possible sharing of the data, biomaterials and imaging data with institutions in countries outside the European Union. This will only be done for the PARIS study.
    - Yes
    - No
- I consent to the possible sharing of the data, biomaterials and imaging data with commercial entities. This will only be done for the PARIS study.
    - Yes
    - No
- I agree with the participation of this person with the study.

For peer review only

English translation of the informed consent form of the PARIS study

Name legal representative:
Date of birth:
Relation to the person:
Signature: Date: __/ __/ __

Declaration of researcher

I hereby declare that I have fully informed the legal representative regarding the biobank Pearl AAA. If information comes to light during the course of the study that could affect the legal representative's consent, I will inform him/her of this in a timely fashion.

Name researcher:
Signature: Date: __/ __/ __

Additional information has been given by (if applicable):
Name:
Role:
Signature: Date: __/ __/ __

Study participants receive the full information brochure, and a signed copy of the informed consent form.

For peer review only

BMJ Open

Design and protocol of a comprehensive multicentre biobank for abdominal aortic aneurysms

Journal:	*BMJ Open*
Manuscript ID	bmjopen-2018-028858.R2
Article Type:	Protocol
Date Submitted by the Author:	21-May-2019
Complete List of Authors:	Jalalzadeh, Hamid; Amsterdam UMC, Surgery<br>Indrakusuma, Reza; Amsterdam UMC, Surgery<br>Blankensteijn, Jan; Amsterdam UMC, Surgery<br>Wisselink, Willem; Amsterdam UMC, Surgery<br>Yeung, Kakkhee; Amsterdam UMC, Surgery<br>Lindeman, Jan; Leiden University Medical Center, Vascular surgery<br>Hamming, Jaap; LUMC, surgery<br>Koelemay, Mark; Amsterdam UMC, Surgery<br>Legemate, Dink; Amsterdam UMC, Surgery<br>Balm, Ron; Amsterdam UMC, Surgery
**Primary Subject Heading**:	Surgery
Secondary Subject Heading:	Cardiovascular medicine
Keywords:	Vascular surgery < SURGERY, VASCULAR SURGERY, Cardiovascular imaging < RADIOLOGY & IMAGING

SCHOLARONE™ Manuscripts

Design and protocol of a comprehensive multicentre biobank for abdominal aortic aneurysms

Hamid Jalalzadeh*[1], Reza Indrakusuma*[1], Jan D. Blankensteijn[2], Willem Wisselink[2], Kak K. Yeung[2], Jan H.N. Lindeman[3], Jaap F. Hamming[3], Mark J.W. Koelemay[1], Dink A. Legemate[1], Ron Balm[1]

* Both authors contributed equally

1. Amsterdam UMC, University of Amsterdam, Department of Surgery, Amsterdam Cardiovascular Sciences, Meibergdreef 9, 1105 AZ, Amsterdam, The Netherlands
2. Amsterdam UMC, Vrije Universiteit Amsterdam, Department of Surgery, Amsterdam Cardiovascular Sciences, De Boelelaan 1117, 1081 HV, Amsterdam, The Netherlands
3. Leiden University Medical Center, Department of Surgery, Albinusdreef 2, 2333 ZA, Leiden, The Netherlands

Current steering committee:

Hamid Jalalzadeh: h.jalalzadeh@amc.uva.nl

Reza Indrakusuma: r.indrakusuma@amc.uva.nl

Jan D. Blankensteijn: j.blankensteijn@vumc.nl

Willem Wisselink: w.wisselink@vumc.nl

Kak. K. Yeung: k.yeung@vumc.nl

Jan H.N. Lindeman: j.h.n.lindeman@lumc.nl

Jaap F. Hamming: j.f.hamming@lumc.nl

Mark. J.W. Koelemay: m.j.koelemaij@amc.uva.nl

Dink A. Legemate: d.a.legemate@amc.uva.nl

Ron Balm: r.balm@amc.nl

For peer review only

Corresponding author and sponsor-investigator:

Ron Balm

r.balm@amc.nl

telephone number: +31205667832

Amsterdam UMC, University of Amsterdam, Department of Surgery, Amsterdam Cardiovascular Sciences, Meibergdreef 9, 1105 AZ, Amsterdam, The Netherlands

Licence per instructions

I, the Submitting Author has the right to grant and does grant on behalf of all authors of the Work (as defined in the below author licence), an exclusive licence and/or a non-exclusive licence for contributions from authors who are: i) UK Crown employees; ii) where BMJ has agreed a CC-BY licence shall apply, and/or iii) in accordance with the terms applicable for US Federal Government officers or employees acting as part of their official duties; on a worldwide, perpetual, irrevocable, royalty-free basis to BMJ Publishing Group Ltd ("BMJ") its licensees and where the relevant Journal is co-owned by BMJ to the co-owners of the Journal, to publish the Work in BMJ Open and any other BMJ products and to exploit all rights, as set out in our licence.

The Submitting Author accepts and understands that any supply made under these terms is made by BMJ to the Submitting Author unless you are acting as an employee on behalf of your employer or a postgraduate student of an affiliated institution which is paying any applicable article publishing charge ("APC") for Open Access articles. Where the Submitting Author wishes to make the Work available on an Open Access basis (and intends to pay the relevant APC), the terms of reuse of such Open Access shall be governed by a Creative Commons licence – details of these licences and which Creative Commons licence will apply to this Work are set out in our licence referred to above.

Word count:

- Abstract: 324
- Manuscript excluding title page, abstract, tables, acknowledgements, contributions and references: 2704

For peer review only

ABSTRACT

Introduction

The pathophysiology and natural course of abdominal aortic aneurysms (AAAs) are insufficiently understood. In order to improve our understanding, it is imperative to carry out longitudinal research which combines biomarkers with clinical and imaging data measured over multiple time points. Therefore, a multicentre biobank, databank and imagebank has been established in the Netherlands: the '*Pearl Abdominal Aortic Aneurysm*' (AAA bank).

Methods and analysis

The AAA bank is a prospective multicentre observational bio-, data- and imagebank of patients with an AAA. It is embedded within the framework of the String of Pearls Institute (PSI) which facilitates uniform biobanking in all university medical centres (UMCs) in the Netherlands. The AAA bank has been initiated by the two UMCs of Amsterdam UMC and by Leiden University Medical Center. Participants will be followed during AAA follow-up. Clinical data are collected every patient contact. Three types of biomaterials are collected at baseline and during follow-up: blood (including DNA and RNA), urine and AAA tissue if open surgical repair is performed. Imaging data that are obtained as part of clinical care are stored in the imagebank. All data and biomaterials are processed and stored in a standardised manner. AAA growth will be based on multiple measurements and will be analysed with a repeated measures analysis. Potential associations between AAA growth and risk factors that are also measured on multiple time points can be assessed with multivariable mixed-effects models, while potential associations between AAA rupture and risk factors can be tested with a conditional dynamic prediction model with landmarking, or with joint models in which linear mixed-effects models are combined with Cox regression.

Ethics and dissemination

The AAA bank is approved by the Medical ethics Board of the Amsterdam UMC (University of Amsterdam).

For peer review only

Registration: this study was retrospectively registered on October 25, 2017 on www.clinicaltrials.gov (NCT03320408).

Keywords:

Abdominal aortic aneurysm, biobank, databank, imagebank, pathophysiology, natural history

For peer review only

Strengths and limitations of this study

- Longitudinal collection of clinical data, biomaterials and imaging data of patients with an abdominal aortic aneurysm provides ample opportunity to better understand the natural history, and to search for prognostic risk factors that might benefit future treatment.
- The inclusion of patients with a small asymptomatic abdominal aortic aneurysm offers the possibility to study natural history from an early stage.
- Standardised collection and storage of biomaterials allows the analysis of patients included at different hospitals.
- Study follow-up is done at routine follow-up appointments to minimise participant burden, yet also means that study follow-up will vary between patients.
- Patient recruitment currently only takes place in university medical centres which are tertiary referral centres.

For peer review only

INTRODUCTION

An abdominal aortic aneurysm (AAA) is a focal dilatation of the abdominal aorta that affects mostly elderly men. The prevalence of AAA in the general population is 1.3-2.2% in 65-year old men.[1, 2] An AAA is an asymptomatic disorder which is associated with a high risk of mortality in case of rupture.[3] Yet, the risk of rupture itself is difficult to measure accurately and also varies considerably between patients. Consequently, the management of patients with an asymptomatic AAA is focused on balancing the risk of rupture with other competing risks of death, with the aim of preserving quality and quantity of life. On the one hand, asymptomatic patients who are estimated to have a high risk of rupture, in general at a diameter of more than 5.5 cm for men, may be offered prophylactic AAA repair if the risk of rupture outweighs any procedural and/or competing risks.[4] On the other hand, asymptomatic patients for whom the risk of rupture is estimated to be smaller than procedural and/or competing risks will be offered surveillance – for example those with an AAA diameter smaller than 5.5 cm or those with severe comorbidities. A large body of research has been dedicated to determining the optimal surgical treatment for AAA, focusing on either the threshold diameter for repair, the method of repair, or the outcome and follow-up after treatment.

Although many studies have tried to unravel AAA pathophysiology, this aspect is still insufficiently understood. Most of the current knowledge originates from histopathological studies that reflect the end stage of AAA disease. Early determinants and drivers of AAA formation are largely unidentified due to the unavailability of AAA tissue from an early stage of the disease. Therefore, the known determinants of AAA development are limited to general risk factors such as male sex, ageing, smoking or connective tissue diseases.[5, 6]

Recent studies focused on biomarker research to find early disease markers and potential targets for pharmacological treatment. Promising circulating biomarkers included markers of matrix turnover (matrix metalloproteinases), markers of inflammation (interleukins and C-reactive protein) and markers of lipid metabolism (lipoproteins).[7-9] Unfortunately, to date, none of these biomarkers have found their way to clinical practice. This is mostly due to their low prognostic value for AAA

For peer review only

progression, and because many studies have not corrected for factors such as smoking or comorbidities.[8] The same applies to new imaging biomarkers such as ^{18}F-fluoro-deoxy-glucose (^{18}F-FDG), or biomechanical markers such as peak wall stress and wall shear stress.[10-12]

To better understand AAA pathophysiology and its natural course, it is imperative to combine studies with biomarkers, imaging markers, and longitudinal data. To that end, a multicentre databank, biobank and imagebank has been established in the Netherlands: the '*Pearl Abdominal Aortic Aneurysm*, hereafter referred to as the AAA bank. The aim of this project is to facilitate future studies on AAA. It is part of the Dutch String of Pearls Institute (PSI), which facilitates uniform biobanking in all eight university medical centres (UMCs) in the Netherlands.[13] The systematic collection of clinical data, biomaterials and imaging data will enable a diverse range of studies on patients with AAA. The AAA bank will especially focus on patients with a small AAA to collect longitudinal data early on in the development of AAA.

The first proposed study that will be carried out with the collected biomaterials is the 'Predicting aneurysm growth and rupture with longitudinal biomarkers' (PARIS) study. The PARIS study aims to determine the association between AAA progression and the evolution of serum levels of proteases and cytokines.

The scientific aims of the AAA bank are (1) to gain insight in the pathophysiology and natural history of AAA; (2) to gain more knowledge about the rupture risk of AAA; and (3) to evaluate and improve treatment of patients with an AAA. Future studies with data from the AAA bank must adhere to these scientific aims.

For peer review only

METHODS AND ANALYSIS

Study design

The AAA bank is a prospective multicentre observational biobank, databank and imagebank of patients with AAA in The Netherlands (ClinicalTrials.gov: NCT03320408, see table 1 for the WHO Trial Registration Data Set).

Table 1. WHO trial registration data set

Primary registry and trial identifying number	ClinicalTrials.gov: NCT03320408
Date of registration in primary registry	25 October 2017
Secondary identifying numbers	NL59991.018.17, PARIS study, biobank Pearl AAA
Sources of monetary of material support	AMC Foundation for monetary support.
Primary sponsor	Academic Medical Center – University of Amsterdam
Secondary sponsor(s)	none
Contact for public queries	Els Kuiters, e.kuiters@amc.uva.nl, +31205667832, Meibergdreef 9, 1105AZ, Amsterdam, the Netherlands.
Contact for scientific queries	Principal investigator: prof. R. Balm, r.balm@amc.nl, +3120-5667832, Meibergdreef 9, 1105AZ, Amsterdam, The Netherlands.<br><br>Els Kuiters, e.kuiters@amc.uva.nl, +31205667832, Meibergdreef 9, 1105AZ, Amsterdam, the Netherlands.
Public title	PARIS study & biobank Pearl AAA
Scientific title	Predicting aneurysm growth and rupture with longitudinal biomarkers (PARIS study) & biobank Pearl AAA
Countries of recruitment	The Netherlands
Health condition(s)	Abdominal aortic aneurysm
Intervention(s)	None
Key inclusion and exclusion criteria	Inclusion criteria: adult with an AAA, or who has been previously treated for an AAA. Adequate comprehension of the Dutch language to provide written informed consent.<br>Exclusion criteria: Decisionally impaired patients. The exception are patients who are decisionally impaired due to the effects of an acute AAA for whom a separate recruitment and consent procedure exists.
Study type	Observational longitudinal patient registry and biobank
Date of first enrolment	4 October 2017
Sample size	Planned: 750<br>Currently enrolled: 87
Recruitment status	Recruiting; participants are currently being recruited and enrolled
Primary outcome(s)	Outcome: AAA growth. Time frame: up to 10 years of follow-up<br>Outcome. AAA rupture. Time frame: up to 10 years of follow-up<br>Outcome: all-cause mortality. Time frame: up to 10 years of follow-up<br>Outcome: evolution of serum levels of proteases and cytokines. Time frame:

For peer review only

	a maximum of 1 measurement annually up to 10 years of follow-up<br>Outcome: proteases and cytokine levels in AAA tissue. Time frame: if open AAA repair is performed and AAA tissue is collected. This is a one-time measurement.
Key secondary outcome(s)	Outcome: incidence and type of complications after AAA repair. Time frame: up to 10 years of follow-up after AAA repair
Ethics review	Status: approved<br>Date of approval: 25 August 2017<br>Name and contact details of ethics committees:<br>Medical Ethics Board of Amsterdam UMC (University of Amsterdam). mecamc@amc.uva.nl, +31205667389, Trinity building C, fourth floor, Pietersbergweg 17, 1105BM Amsterdam, the Netherlands.<br><br>Biobank Ethics Board of Amsterdam UMC (University of Amsterdam), biobanktoetsing@amc.uva.nl, +31205666730, Meibergdreef 9, 1105AZ, Amsterdam, the Netherlands.
Completion date	Expected: 4 October 2032
Summary results	No results yet.
IPD sharing statement	Plan to share IPD: Yes<br>Plan description: IPD sharing for research will be allowed in a data sharing procedure. Scientific requests need to fall under the scope of the scientific aims as formulated in this manuscript. Researchers willing to requests IPD can initiate this procedure by contacting the researchers. Data will be released depending on the scientific quality of the submitted request.

AAA = abdominal aortic aneurysm

The active protocol at the time of writing is version 3, 22 December 2017. The AAA bank is embedded within the framework of PSI, which is co-financed by the Dutch government and the Netherlands Federation of University Medical centres (NFU).[13] PSI was established in 2007 and aims to facilitate standardised nationwide biobanks and clinical databases.[14] At the time of writing, 17 different patient cohorts (*Pearls*) for different diseases have been initiated within the PSI framework. Examples include the Diabetes Pearl, the Pearl Neurodegenerative Diseases, the Stroke Pearl, and the Dutch Pancreas biobank.[15-18] All these biobanks adhere to an internal regulatory framework, which prescribes legal and ethical rules concerning the conduct of all Pearl-related activities.[14]

The AAA bank has been initiated by the two UMCs from Amsterdam University Medical Centers (University of Amsterdam and Vrije Universiteit Amsterdam) and by Leiden University Medical Center (LUMC). In accordance with the goals of PSI and NFU, the AAA bank aims to expand to the other Dutch UMCs. The AAA bank is currently an ongoing project, with active recruitment and collection of data and biomaterials. The first patient was recruited on October 4, 2017.

For peer review only

Study population

All capable adults with AAA in participating university medical centres are eligible for inclusion in the AAA bank. This also includes patients who previously have undergone AAA repair. Patients who are incapable due to a ruptured AAA (RAAA) can also be included, using a special recruitment process, which will be described below. All included patients will be followed for as long as they visit their treating vascular surgeon.

Recruitment procedure

Eligible patients are recruited at the inpatient or outpatient clinic of the department of vascular surgery of the participating hospitals by research physicians and/or data managers. Participants who agree to participate give written informed consent during their visit to the inpatient or outpatient clinic. When patients arrive in an emergency setting with a ruptured or symptomatic AAA, oral consent is required from either the patient or a legal representative. This oral consent has to be confirmed in writing at a later stage – either by the patient, or by a representative in case of a fatal outcome. In the event that no written informed consent can be obtained, all data and biomaterials collected for the AAA bank will be destroyed.

Study procedures

The study procedures of the AAA bank are embedded within regular AAA treatment and are carried out by physician-researchers and data managers. Standard operating procedures have been set up to minimise the amount of missing data. Clinical data and biomaterials are collected during and after visits that are already part of clinical treatment, to limit participant burden and improve participant retention. No research visits will be planned for the AAA bank. In addition, only imaging data that is obtained as part of clinical care will be stored in the AAA bank.

For peer review only

In line with regular AAA management, four distinct study phases have been identified: inclusion phase, surveillance phase, surgical phase, and postoperative phase (Figure 1; blue boxes). Within each phase, multiple visits can take place – especially in the surveillance or postoperative phase. These phases can theoretically continue indefinitely, depending on the course of the AAA. With regard to the 'surveillance phase', the Dutch AAA guideline advises that patients visit a vascular surgeon at intervals of two years, one year, or three months, depending on the AAA diameter.[19] Patients can move from the 'surveillance phase' to the 'surgical phase' and subsequently to the 'postoperative phase' (Figure 1). Furthermore, there will be patients who at some point reach the threshold diameter for repair, yet who do not undergo repair for various reasons such as severe comorbidity. In these cases, an individual clinical decision – unrelated to their participation with the AAA bank – will have to be made together with the patient whether surveillance continues with regular intervals or whether the patient chooses to quit with surveillance altogether. Patients who choose to continue with surveillance will still be asked for biomaterials and clinical data, while patients who quit surveillance can be analysed using the previously collected data up until that moment. Furthermore, the latter patients can also be included in survival analyses as mortality data can be sourced from either the municipal registry of persons or their general practitioner.

Clinical data – PRISMA

All clinical data are collected according to an information model called Parelsnoer Repository for Information Specification, Modelling and Architecture (PRISMA), which was constructed with the assistance of an experienced information architect of PSI. The majority of clinical data will be collected from electronic health records (EHRs) in order to reduce participant burden, while only a minority of the data will be collected through questionnaires, as outlined in table 2.

For peer review only

Table 2. The PRISMA information model of the AAA bank

<table>
<tr><th>eDCS name</th><th colspan="3">Inclusion</th><th colspan="3">Follow-up[N]</th><th>Stream</th><th>Source</th></tr>
<tr><th></th><th>Asymptomatic AAA</th><th>Operation for acute AAA</th><th>Previously operated AAA</th><th>Surveillance phase</th><th>Surgical phase</th><th>Postoperative phase</th><th></th><th></th></tr>
<tr><td>Patient information</td><td colspan="3">1</td><td colspan="3">M</td><td></td><td>EHR</td></tr>
<tr><td>Informed consent</td><td colspan="3">1</td><td colspan="3">M</td><td></td><td>n.a.</td></tr>
<tr><td>Living situation</td><td colspan="3">1</td><td colspan="3">1</td><td></td><td>Q</td></tr>
<tr><td>Current aneurysm state</td><td colspan="3">1</td><td colspan="3">1</td><td></td><td>EHR</td></tr>
<tr><td>First occurrence comorbidity</td><td colspan="3">1</td><td colspan="3">M</td><td></td><td>EHR</td></tr>
<tr><td>Family history</td><td colspan="3">0..n</td><td colspan="3">M</td><td></td><td>Q</td></tr>
<tr><td>Social history</td><td colspan="3">1</td><td colspan="3">M</td><td></td><td>Q</td></tr>
<tr><td>Initial aneurysm characteristics</td><td colspan="3">1</td><td colspan="3"></td><td></td><td>EHR</td></tr>
<tr><td>Comorbidity status</td><td colspan="3">1</td><td colspan="3">1</td><td></td><td>EHR</td></tr>
<tr><td>Medication</td><td colspan="3">1</td><td colspan="3">1</td><td></td><td>EHR, Q</td></tr>
<tr><td>Intoxications</td><td colspan="3">1</td><td colspan="3">1</td><td></td><td>EHR, Q</td></tr>
<tr><td>Physical examination</td><td colspan="3">1</td><td>1</td><td>1</td><td></td><td></td><td>EHR, Q</td></tr>
<tr><td>Blood test results</td><td colspan="3">1</td><td colspan="3">1</td><td></td><td>EHR</td></tr>
<tr><td>Surveillance</td><td>1</td><td colspan="2"></td><td>1</td><td colspan="2"></td><td></td><td>EHR</td></tr>
<tr><td>Preoperative assessment</td><td></td><td colspan="2">1</td><td></td><td>1</td><td></td><td></td><td>EHR</td></tr>
<tr><td>AAA repair</td><td></td><td colspan="2">1</td><td></td><td>1</td><td></td><td></td><td>EHR</td></tr>
<tr><td>Postoperative admission</td><td></td><td colspan="2">1</td><td></td><td>1</td><td></td><td></td><td>EHR</td></tr>
<tr><td>Postoperative follow-up</td><td></td><td colspan="2">1</td><td></td><td></td><td>1</td><td></td><td>EHR</td></tr>
</table>

For peer review only

For peer review only - http://bmjopen.bmj.com/site/about/guidelines.xhtml

Biomaterials	1	1		n.a.
Cardiovascular events			dt	EHR
Other surgical procedures			dt	EHR
Malignancies			dt	EHR
AAA imaging	0..n		dt	EHR
Complications			dt	EHR
Imaging data	1	1	dt	EHR
	eDCS: electronic Data Capture System;<br>1: indicates that an eDCS is registered once and is overwritten in case of future changes.<br>M: Means 'Modify', and indicates that the eDCS can be modified within the same observation period. N: Indicates that this eDCS can be registered multiple times (but at least once)<br>0..n: Indicates that this eDCS can be registered multiple times (but is not mandatory)<br>The eDCS from previous observation periods remain unchanged.<br>dt: the eDCS is saved as a time-stream with date-time format.<br>EHR: electronic health records<br>Q: questionnaire			

For peer review only

Furthermore, PRISMA consists of electronic Data Capture Systems (eDCSs), with each eDCS covering a certain theme, as described in more detail in Table 3.

Table 3. General description of each eDCS in PRISMA

eDCS	General description
Patient information	Study number, year of birth and survival status including cause of death.
Informed consent	Status of informed consent (given or withdrawn), date of informed consent, individual patient decisions with regard to additional consent options.
Living situation	e.g. independent or assisted living.
Current aneurysm state	e.g. asymptomatic, acute or postoperative status.
First occurrence comorbidity	This eDCS contains a predefined list of comorbidities. Only if a participant has any of these comorbidities will this be registered, including the initial date of diagnosis.<br><br>The list includes:<br>Hypertension, COPD, idiopathic lung fibrosis, peptic ulcer, diabetes mellitus, hypo- and hyperthyroidism, hepatitis, portal hypertension, liver cirrhosis, any other liver disease, peripheral arterial disease, intracranial aneurysm, popliteal aneurysm, any other aneurysm, connective

For peer review only

	tissue disease, hypercholesterolaemia, carotid artery disease, renal dysfunction, renal disorders, ischaemic heart disease, heart failure, heart arrhythmia, heart valve disorders, any other cardiac disorder, hemi- or paraplegia, dementia and AIDS.<br><br>Free text descriptions can be provided for cases with an “any other” comorbidity as mentioned above.
Family history	Registration of each known family member with (a history of) aortic aneurysmal disease and if known, at what age and whether there was an aortic rupture.
Social history	Marital status, employment status, educational status.
Initial aneurysm characteristics	Pathogenesis and type of AAA, status of AAA at time of inclusion, date of initial diagnosis, shape of AAA.
Comorbidity status	Status of a predefined list of comorbidities. This will be registered if this is known at the time of a visit to the department of vascular surgery.<br><br>The list includes:<br>COPD, diabetes mellitus (e.g. dependency on

For peer review only

	insulin), peripheral arterial disease (e.g. Fontaine classification), renal dysfunction, angina pectoris, heart failure.
Medication	This eDCS contains a predefined list of medications. Only pills and injections will be registered. If a medication is not included in the list, it will be added via a free text description. Dosage will be registered for cardiovascular medications.<br><br>The list includes:<br>All types of cardiovascular medications, diabetes mellitus medication, corticosteroids, immunosuppressive medication and benzodiazepines.
Intoxications	Nicotine and alcohol use.
Physical examination	Body weight and patient length.
Blood test results	This eDCS contains a predefined list of blood tests, which will be registered if these have been carried out for clinical care.<br><br>The list includes:<br>Creatinine, estimated glomerular filtration rate, total cholesterol, high-density lipoprotein cholesterol, low-density lipoprotein cholesterol,

For peer review only

	triglycerides,
Surveillance	Date of surveillance visit, treatment plan including motivation.
Preoperative assessment	American Society of Anaesthesiologists physical status classification
AAA repair	Date of AAA repair, type of AAA repair, intraoperative complications
Postoperative admission	Length of hospitalisation, intensive care hospitalisation
Postoperative follow-up	Date of postoperative follow-up visit, treatment plan including motivation
Cardiovascular events	Myocardial infarction, cerebrovascular accident, thromboembolic events including dates.
Other surgical procedures	This eDCS contains a predefined list of certain procedures.<br><br>The list includes:<br>Cardiac surgery, vascular surgery, abdominal and thoracic surgery, and transplant surgery.
Malignancies	General type of malignancy, date of diagnosis, type of treatments.
AAA imaging	Date and type of AAA imaging. AAA status at time of imaging (e.g. asymptomatic, acute or postoperative), AAA diameter,
Complications	Complications after any AAA repair will be

For peer review only

	registered according to a predefined code list of the Dutch National Surgical Complications Registry (LHCR), which was established by a committee of the Association of Surgeons in the Netherlands (NVvH).

Data are registered via local data capture platforms, such as Castor EDC[20] (Ciwit, the Netherlands, which is hosted by True[21] in the Netherlands), and are centrally stored in Project Manager Internet Server (ProMISe)[22], a web-based relational database management system (Advanced Data Management, the Netherlands). These systems are compliant with Good Clinical Practice and are ISO27001 certified. All patients are being registered under a study number that is electronically assigned by a designated tool. This study number is used during data collection and data processing.

Biomaterials

Three types of biomaterials are collected: blood, urine and AAA tissue (Table 4).

Table 4. Biomaterials collected for the AAA bank

Biomaterials	Inclusion	Surveillance phase	Surgical phase	Postoperative phase	Storage temperature
Blood - EDTA plasma	once	max. 1 / year	once	Once: 1 year postoperative	≤ -80°C
Blood - Serum	once	max. 1 / year	once	Once: 1 year postoperative	≤ -80°C
Blood - Citrate plasma	once	max. 1 / year	once	Once: 1 year postoperative	≤ -80°C
Blood - EDTA for DNA	once				4°C or ≤ -20°C
Blood - PAXgene for RNA	once				≤ -80°C
Urine	once	max. 1 / year	once	Once: 1 year postoperative	≤ -80°C
Aneurysm tissue - frozen			once		≤ -80°C
Aneurysm tissue – FFPE			once		room temperature

EDTA = Ethylenediaminetetraacetic acid, FFPE = Formalin-fixed paraffin-embedded

For peer review only

Blood and urine are collected repeatedly during the surveillance phase, up to a maximum of once per year to limit patient burden. Blood is saved as plasma, serum and whole blood for DNA and RNA. When open surgical repair of AAA is performed, aortic tissue is collected, snap frozen and stored at -80°C, and as formalin-fixed paraffin-embedded (FFPE) tissue. After surgery for AAA, blood and urine are collected up to one year postoperatively (Table 4). All procedures concerning biomaterials adhere to standard operating procedures outlined by PSI. Furthermore, all biomaterials are stored in designated PSI biobanks within each UMC. Thus, all biomaterials are stored in the UMC where they are collected.

Imaging data

In order to facilitate future imaging studies, computed tomography (CT) and magnetic resonance imaging (MRI) data that are obtained as part of clinical care are also stored centrally. At the time of writing, the participating centres are harmonizing the CT protocols to acquire standardised images. Partners currently involved in the central storage of images are Translational Research IT (TRaIT, part of Dutch non-profit organisation Lygature) and Vancis, a Dutch service provider of IT services for research with a ISO27001 certificate.[23-25] The imaging data are stored centrally in an Extensible Neuroimaging Archive Toolkit (XNAT) server (Buckner Lab, Harvard University, United States of America).[26] This server is operated by TraIT and is hosted by Vancis.

Imaging data are stored as Digital Imaging and Communications in Medicine (DICOM) data. Before the data are sent to the central server, the study number is allocated to the imaging data to enable linking of imaging data with clinical data and biomaterials. All other identifiable data are removed from the DICOM data using Clinical Trial Processor (CTP, Radiological Society of North America, United States of America).[27]

Statistical analyses

For peer review only

The primary outcomes are AAA growth, AAA rupture and all-cause mortality. AAA growth will be based on multiple measurements and will therefore be analysed with a repeated measures analysis. Potential associations between AAA growth and risk factors that are also measured on multiple time points can be assessed with multivariable mixed-effects models, while potential associations between AAA rupture and risk factors can be tested with a conditional dynamic prediction model with landmarking, or with joint models in which linear mixed-effects models are combined with Cox regression.

Due to the expected multifactorial aspect of AAA growth, even weak correlations are of interest to detect. To that end, at least 750 patients are required to be able to detect a correlation coefficient of 0.16 with a power of 80%, and a significance level of .05.

Patient and public involvement

This research was done without patient involvement. Patients were not invited to comment on the study design and were not consulted to develop patient relevant outcomes or interpret the results. Patients were not invited to contribute to the writing or editing of this document for readability or accuracy.

ETHICS AND DISSEMINATION

The Medical Ethics Board and the Biobank Ethics Board of Amsterdam UMC (University of Amsterdam) have approved the AAA bank together with the PARIS study (Table 5) within the scope of the Dutch Medical Research Involving Human Subjects Act (WMO) under registration number NL59991.018.17.

For peer review only

Table 5. The PARIS study, the first proposed study that will be facilitated by the AAA bank

Aims	The aims of the PARIS study are: 1) to determine the association between AAA progression (growth or rupture) and the evolution of serum levels of both proteases and cytokines, 2) to determine the association between overall survival and the evolution of serum levels of proteases and cytokines, 3) to determine the association between serum levels of proteases and cytokines and level of proteases and cytokines in AAA tissue, and finally 4) to determine the incidence of, and characterise the type of complications after AAA repair.
Outcomes	The primary outcomes are: AAA growth, AAA rupture, all-cause mortality, serum levels of cytokines and proteases, and cytokine and protease levels in AAA tissue. Secondary outcomes are the incidence and type of complications after AAA repair.
Sample Size	Because of the multifactorial aspect of AAA progression, we calculated a sample size that will be sufficient for detecting weak correlations between cytokine and protease levels and AAA growth. To detect a correlation coefficient of 0.16 with a power of 80%, and a significance level of .05, a sample size of 750 participants is required. Strategies to achieve sufficient participant enrolment include simple eligibility criteria, and the expressed intention to extend the number of participating centres.
Statistical analysis	Categorical variables will be presented as numbers and percentages, and will be compared between groups with the Chi-square test of Fisher exact test where appropriate. Continuous variables will be presented as means ± standard deviation or as medians with the interquartile range, depending on distribution. Distribution of continuous variables will be tested with the Shapiro-Wilk test. Continuous variables will be compared between groups with either the unpaired t-test or the Mann–Whitney *U* test depending on distribution.<br><br>Individual AAA growth (based on repeated AAA diameter measurements) and the evolution of serum levels of cytokines and proteases will each be analysed with linear mixed-effects models to estimate the slope of temporal change (i.e. the evolution through time). The linear mixed-effects model that estimates the slope for the evolution of each biomarker will also adjust for potentially relevant covariables including but not limited to sex, age and cardiovascular comorbidity. A forward stepwise selection will be used to select only those covariables that were significant in univariable analysis. Finally, correlations coefficient will be estimated between the slope of AAA progression and the slope of each biomarker.<br><br>Freedom from AAA rupture and all-cause mortality will be estimated with the Kaplan-Meier method, while joint modelling will be used to assess the association between the evolution of serum levels of proteases and cytokines and AAA rupture and all-cause mortality. A joint model will be performed per biomarker. In order to assess the association, joint modelling combines linear mixed-effects models for the estimation of slope of temporal change per biomarker with Cox regression for the analysis of freedom

	from AAA rupture and all-cause mortality, in order to estimate hazard ratios. The estimated slope and value of the studied biomarker will be added as covariables in each joint model. Furthermore, both models will be adjusted for potentially relevant covariables, including but not limited to sex, age, most recent AAA diameter and cardiovascular comorbidity using the same selection procedure. In addition, AAA growth instead of the most recent AAA diameter will be analysed in a separate multivariable joint model. In all analyses a p value $< .05$ will be considered statistically significant, including the covariable selection procedure.

In general, biobanks in the Netherlands do not fall within the scope of the WMO. To be eligible for assessment under WMO, the formulation of a specific research question is required. However, specific research questions are often not present at the moment of biobank initiation.[28] Yet, the submission of the AAA bank together with the PARIS study (that contains a specific research question) enabled approval of the combined project within the scope of the WMO. This approval ensures that the AAA bank adheres to the highest legal and medical-ethical standards, and that participation of other future centres can be realised using the existing procedures of the WMO. Because of this design, all participating patients sign two informed consent forms – one for the biobank and one for the PARIS study (see appendix 1-6 for English versions of the forms). By consenting to the AAA bank and signing its consent form, patients not only consent to the collection and storage of their biomaterials and data, but also to future analyses of it for research about AAA. Any significant modification to the protocol which may impact patient safety, or the conduct, design or analysis of the study requires formal amendment to the protocol. These will need to be approved by the Medical Ethics Board and the Biobank ethics Board of Amsterdam UMC (University of Amsterdam).

To accommodate patients with different views on data collection, participants can refuse collection of DNA and the sharing of their data with foreign and/or commercial parties. All collected data and biomaterials will be stored for a maximum of 50 years. When a participant decides to withdraw from the AAA bank, all stored biomaterials and data will be destroyed or deleted. When reasonably possible, this is also done with materials that are sent out for a specific study.

For peer review only

Scientific board for future studies

Collected data and biomaterials of the AAA bank can only be used for future studies that fall within the scope of the scientific aims of the AAA bank, and that are approved by the scientific board. Researchers can submit a study proposal with the scientific board of the AAA bank. Each study proposal must include a study objective and/or research questions, the type of data and biomaterials required, a statistical analysis plan and an agreed upon authorship policy. This board oversees all requests for data and consists of five members, with a minimum of one biostatistician (A.H. Zwinderman, Amsterdam UMC, University of Amsterdam, Department of Clinical Epidemiology) and either a legal expert or ethicist (provided by the String of Pearls Institute) among its members. The other three members are currently vascular surgeons from the initiating UMCs (DAL, WW, JFH). If a study proposal is approved by the scientific board, subsequent medical-ethical approval will be acquired if required by Dutch law or local guidelines. Furthermore, data is only released in accordance with standard PSI procedures.

Results of individual studies will be published in peer-reviewed scientific journals and will be presented at international conferences.

For peer review only

Acknowledgements

We thank the String of Pearls Institute for their help in initiating and designing the AAA bank and the PARIS study. In addition, we would like to thank Roger Snijder, information architect of PSI, for his help in designing our information model.

For peer review only

References

1. Svensjo S, Bjorck M, Gurtelschmid M, Djavani Gidlund K, Hellberg A, Wanhainen A. Low prevalence of abdominal aortic aneurysm among 65-year-old Swedish men indicates a change in the epidemiology of the disease. Circulation. 2011;124:1118-23.
2. Oliver-Williams C, Sweeting MJ, Turton G, Parkin D, Cooper D, Rodd C, et al. Lessons learned about prevalence and growth rates of abdominal aortic aneurysms from a 25-year ultrasound population screening programme. Br J Surg. 2018;105:68-74.
3. Reimerink JJ, van der Laan MJ, Koelemay MJ, Balm R, Legemate DA. Systematic review and meta-analysis of population-based mortality from ruptured abdominal aortic aneurysm. Br J Surg. 2013;100:1405-13.
4. Wanhainen A, Verzini F, Van Herzeele I, Allaire E, Bown M, Cohnert T, et al. Editor's Choice - European Society for Vascular Surgery (ESVS) 2019 Clinical Practice Guidelines on the Management of Abdominal Aorto-iliac Artery Aneurysms. Eur J Vasc Endovasc Surg. 2019;57:8-93.
5. Nordon IM, Hinchliffe RJ, Loftus IM, Thompson MM. Pathophysiology and epidemiology of abdominal aortic aneurysms. Nat Rev Cardiol. 2011;8:92-102.
6. Brown LC, Powell JT. Risk factors for aneurysm rupture in patients kept under ultrasound surveillance. UK Small Aneurysm Trial Participants. Ann Surg. 1999;230:289-96; discussion 96-7.
7. Hellenthal FA, Buurman WA, Wodzig WK, Schurink GW. Biomarkers of AAA progression. Part 1: extracellular matrix degeneration. Nat Rev Cardiol. 2009;6:464-74.
8. Stather PW, Sidloff DA, Dattani N, Gokani VJ, Choke E, Sayers RD, et al. Meta-analysis and meta-regression analysis of biomarkers for abdominal aortic aneurysm. Br J Surg. 2014;101:1358-72.
9. Deeg MA, Meijer CA, Chan LS, Shen L, Lindeman JH. Prognostic and predictive biomarkers of abdominal aortic aneurysm growth rate. Curr Med Res Opin. 2016;32:509-17.
10. Jalalzadeh H, Indrakusuma R, Planken RN, Legemate DA, Koelemay MJ, Balm R. Inflammation as a Predictor of Abdominal Aortic Aneurysm Growth and Rupture: A Systematic Review of Imaging Biomarkers. Eur J Vasc Endovasc Surg. 2016;52:333-42.
11. Indrakusuma R, Jalalzadeh H, Planken RN, Marquering HA, Legemate DA, Koelemay MJ, et al. Biomechanical Imaging Markers as Predictors of Abdominal Aortic Aneurysm Growth or Rupture: A Systematic Review. Eur J Vasc Endovasc Surg. 2016;52:475-86.
12. Chaikof EL, Dalman RL, Eskandari MK, Jackson BM, Lee WA, Mansour MA, et al. The Society for Vascular Surgery practice guidelines on the care of patients with an abdominal aortic aneurysm. J Vasc Surg. 2018;67:2-77 e2.
13. Manniën J, Ledderhof T, Verspaget HW, Snijder RR, Flikkenschild EF, van Scherrenburg NPC, et al. The Parelsnoer Institute: A National Network of Standardized Clinical Biobanks in the Netherlands. Open Journal of Bioresources. 2017;4.
14. Talmon JL, Ros MG, Legemate DA. PSI: The Dutch Academic Infrastructure for shared biobanks for translational research. Summit Transl Bioinform. 2008;2008:110-4.
15. Nederkoorn PJ, van Dijk EJ, Koudstaal PJ, Luijckx GJ, van Oostenbrugge RJ, Visser MC, et al. The Dutch String-of-Pearls Stroke Study: protocol of a large prospective multicenter genetic cohort study. Int J Stroke. 2015;10:120-2.
16. Aalten P, Ramakers IH, Biessels GJ, de Deyn PP, Koek HL, OldeRikkert MG, et al. The Dutch Parelsnoer Institute--Neurodegenerative diseases; methods, design and baseline results. BMC Neurol. 2014;14:254.
17. Strijker M, Gerritsen A, van Hilst J, Bijlsma MF, Bonsing BA, Brosens LA, et al. The Dutch Pancreas Biobank Within the Parelsnoer Institute: A Nationwide Biobank of Pancreatic and Periampullary Diseases. Pancreas. 2018;47:495-501.
18. van't Riet E, Schram MT, Abbink EJ, Admiraal WM, Dijk-Schaap MW, Holleman F, et al. The Diabetes Pearl: Diabetes biobanking in The Netherlands. BMC Public Health. 2012;12:949.
19. Heelkunde NVv. Richtlijn Diagnostiek en behandeling van het aneurysma van de abdominale aorta. 2009.

For peer review only

20. Castor EDC. www.castoredc.com. Accessed June 1, 2018.
21. True. www.true.nl. Accessed June 1, 2018.
22. Project Manager Internet Server (ProMISe). www.msbi.nl/promise/. Accessed June 1, 2018.
23. Translational Research IT (TraIT). www.ctmm-trait.nl/. Accessed June 1, 2018.
24. Lygature. www.lygature.org/. Accessed June 1, 2018.
25. Vancis. www.vancis.nl/. Accessed June 1, 2018.
26. Marcus DS, Olsen TR, Ramaratnam M, Buckner RL. The Extensible Neuroimaging Archive Toolkit: an informatics platform for managing, exploring, and sharing neuroimaging data. Neuroinformatics. 2007;5:11-34.
27. Radiological Society of North America. www.rsna.org/ctp.aspx. Accessed June 1, 2018.
28. Hendriks AC, van Hellemondt RE. Regulating Privacy and Biobanks in the Netherlands. J Law Med Ethics. 2016;44:68-84.

For peer review only

Authors' contributions

HJ and RI contributed equally to this manuscript. All authors (HJ, RI, JDB, WW, KKY, JHNL, JFH, MJWK, DAL, RB) actively contributed to the study conception, design and its protocol. All authors read and contributed to the writing of the manuscript, and approved the final manuscript.

Funding

The AAA bank is funded by the AMC Foundation, which was not involved in the study design, data collection, or writing of this manuscript. Furthermore, the AMC Foundation will not have any role during its execution, analyses, interpretation of the data, or decision to submit results of this and any future study facilitated by the AAA bank.

For peer review only

Competing interests

The authors declare that they have no competing interests.

Data availability statement

Deidentified clinical data, biomaterials and imaging data are available on reasonable request, as outlined in the main text under ‘scientific board for future studies’.

Roles and responsibilities

Principal investigator (RB): final responsibility with regard to design, conduct and protocol (including future revisions) of the AAA bank and the PARIS study. Organises steering committee meetings.

Steering committee (see title page for current members): Agreement of final protocol, recruitment of patients. Reviewing progress of study and if necessary agreeing changes to the protocol to facilitate smooth running of the study.

Lead investigators: in each participating centre a lead investigator (vascular surgeon) has been identified, and is responsible for identification, recruitment, data collection, study follow-up and adherence to the study protocol. Lead investigators are steering committee members.

Trial management committee (principal investigator, research physician and/or data manager): study planning, organising steering committee meetings. Responsible for trial master file.

Since the AAA bank is an observational cohort without any study interventions, a data monitoring committee was not established.

For peer review only

Figures

1. Figure 1. Flow chart of study phases

For peer review only

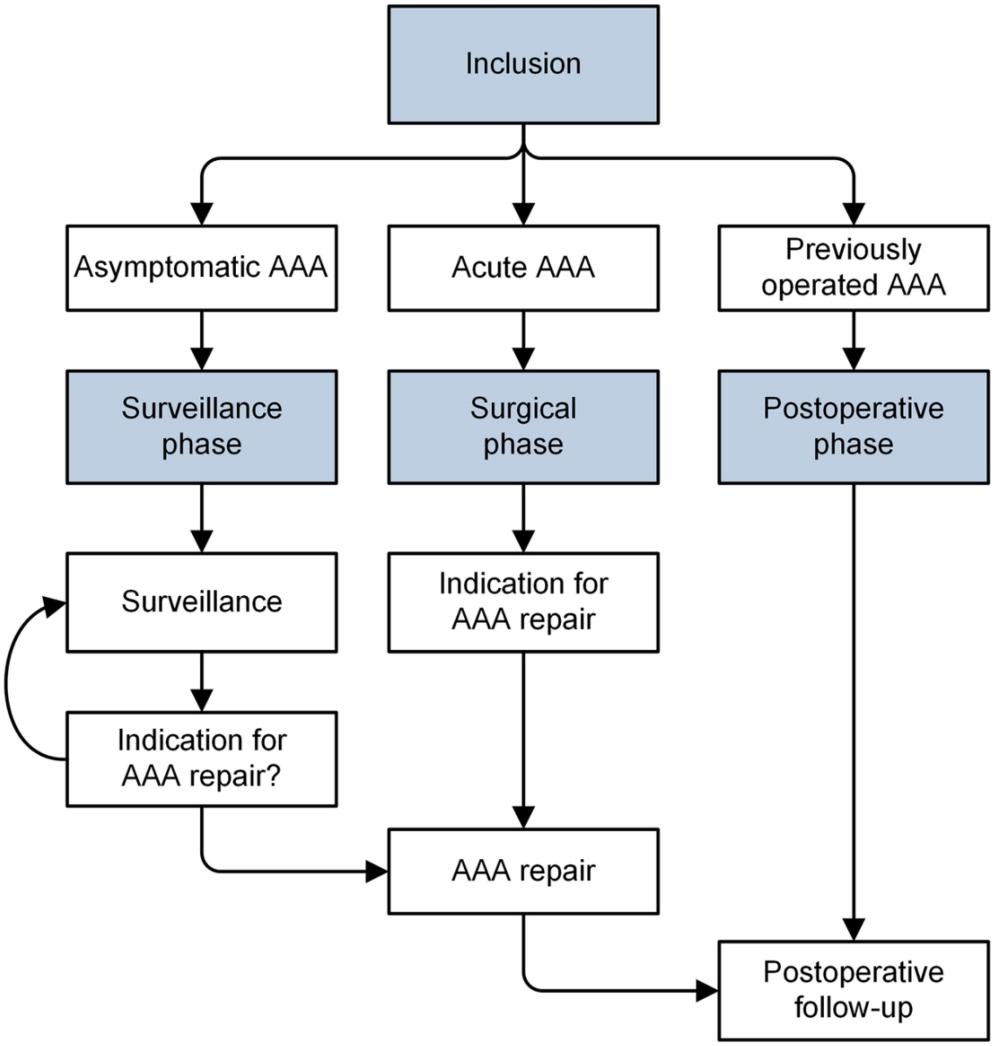

Figure 1. Flow chart of study phases

106x110mm (300 x 300 DPI)

English translation of the informed consent form of the biobank Pearl AAA

Appendix 1. Informed consent form for patients who have an asymptomatic AAA or who have previously undergone AAA repair

Biobank Pearl AAA.

- I consent to the collection, storage and analysis of blood, urine and aortic tissue (in case of open surgery) and data regarding research for abdominal aortic aneurysms.
- I consent to the coded storage of my data for a maximum of 50 years in the biobank Pearl AAA.
- I consent to the coded storage of my biomaterials for a maximum of 50 years for use in future biomedical research.
- I give permission for my GP to be informed that I am participating in this study.
- I give permission for information regarding my health status to be requested from my GP, pharmacy and the municipal registry of persons, under the condition that my privacy will be safeguarded.
- During biomedical research with my biomaterials, incidental findings may be found that are associated with a disease or disorder. Sometimes it only concerns an increased risk. I consent that I will be informed about incidental findings if these are relevant to my (or my family's) health status because either prevention or treatment is possible.
- I consent to the possible sharing of my data, biomaterials and imaging data with non-commercial institutions in countries in the European Union. This will only be done for research purposes.

Additional consent options:

- I consent to the acquisition of my data from national registries concerning the quality of healthcare and its improvement, under the condition that my privacy will be safeguarded.
  - Yes
  - No
- In the event of my death, I consent to the possibility that information regarding my death may be acquired from the Central Bureau of Statistics, under the condition that my privacy will be safeguarded.
  - Yes
  - No
- I consent to the possible sharing of my data, biomaterials and imaging data with institutions in countries outside the European Union. This will only be done for research purposes.
  - Yes
  - No
- I consent to the possible sharing of my data, biomaterials and imaging data with commercial entities. This will only be done for research purposes.
  - Yes

For peer review only

English translation of the informed consent form of the biobank Pearl AAA

- ○ No

- I consent to the study of my DNA for findings associated with the origin and development of abdominal aortic aneurysms and to the effects and complications of medication
  - ○ Yes
  - ○ No

- I consent that I may be approached for additional information and/or biomaterials if this is necessary for a specific biomedical study
  - ○ Yes
  - ○ No

Name participant:
Date of birth:
Signature: Date: __/ __/ __

Declaration of researcher

I hereby declare that I have fully informed this study participant regarding the biobank Pearl AAA. If information comes to light during the course of the study that could affect the study subject's consent, I will inform him/her of this in a timely fashion.

Name researcher:
Signature: Date: __/ __/ __

Additional information has been given by (if applicable):
Name:
Role:
Signature: Date: __/ __/ __

Study participants receive the full information brochure, and a signed copy of the informed consent form.

For peer review only

English translation of the informed consent form of the PARIS study

Appendix 2. Informed consent form for patients who have an asymptomatic AAA or who have previously undergone AAA repair

PARIS study

- I have read the subject information form. I was also able to ask questions. My questions have been answered to my satisfaction. I had enough time to decide whether to participate.
- I know that participation is voluntary. I know that I may decide at any time not to participate after all or to withdraw from the study. I do not need to give a reason for this.
- I give permission for my GP to be informed that I am participating in this study.
- I give permission for information to be requested from my GP or pharmacy regarding my health status.
- I know that some people may have access to all my data to verify the study. These people are listed in this information sheet. I consent to the inspection by them.
- I consent to the collection and use of my data, biomaterials and imaging data as has been specified in this information sheet.
- I consent to the storage of my data for the PARIS study for a maximum of 50 years in the biobank Pearl AAA.
- I consent to the possible sharing of my data, biomaterials and imaging data with non-commercial institutions in countries in the European Union. This will only be done for the PARIS study if necessary.

Additional consent options:

- I consent to the possible sharing of my data, biomaterials and imaging data with institutions in countries outside the European Union. This will only be done for the PARIS study if necessary.
    - Yes
    - No
- I consent to the possible sharing of my data, biomaterials and imaging data with commercial entities. This will only be done for the PARIS study if necessary.
    - Yes
    - No
- I consent to being contacted again after this study for a follow-up study.
    - Yes
    - No
- I want to participate in this study.

For peer review only

English translation of the informed consent form of the PARIS study

Name participant:
Date of birth:
Signature: Date: __/ __/ __

Declaration of researcher

I hereby declare that I have fully informed this study participant regarding the PARIS study. If information comes to light during the course of the study that could affect the study subject's consent, I will inform him/her of this in a timely fashion.

Name researcher:
Signature: Date: __/ __/ __

Additional information has been given by (if applicable):
Name:
Role:
Signature: Date: __/ __/ __

Study participants receive the full information brochure, and a signed copy of the informed consent form.

For peer review only

English translation of the informed consent form of the biobank Pearl AAA

Appendix 3. Informed consent form for patients after emergency AAA repair.

Biobank Pearl AAA

- I consent to the collection, storage and analysis of blood, urine and aortic tissue (in case of open surgery) and data regarding research for abdominal aortic aneurysms.
- I consent to the coded storage of my data for a maximum of 50 years in the biobank Pearl AAA.
- I consent to the coded storage of my biomaterials for a maximum of 50 years for use in future biomedical research.
- I give permission that my GP to be informed that I am participating in this study.
- I give permission for information regarding my health status to be requested from my GP, pharmacy and the municipal registry of persons, under the condition that my privacy will be safeguarded.
- During biomedical research with my biomaterials, incidental findings may be found that are associated with a disease or disorder. Sometimes it only concerns an increased risk. I consent that I will be informed about incidental findings if these are relevant to my (or my family's) health status because either prevention or treatment is possible.
- I consent to the possible sharing of my data, biomaterials and imaging data with non-commercial institutions in countries in the European Union. This will only be done for research purposes.

- I consent to the acquisition of my data from national registries concerning the quality of healthcare and its improvement, under the condition that my privacy will be safeguarded.
    - Yes
    - No
- In the event of my death, I consent to the possibility that information regarding my death may be acquired from the Central Bureau of Statistics, under the condition that my privacy will be safeguarded.
    - Yes
    - No
- I consent to the possible sharing of my data, biomaterials and imaging data with institutions in countries outside the European Union. This will only be done for research purposes.
    - Yes
    - No
- I consent to the possible sharing of my data, biomaterials and imaging data with commercial entities. This will only be done for research purposes.
    - Yes
    - No

For peer review only

English translation of the informed consent form of the biobank Pearl AAA

- I consent to the study of my DNA for findings associated with the origin and development of abdominal aortic aneurysms and to the effects and complications of medication
  - Yes
  - No

- I consent that I may be approached for additional information and/or biomaterials if this is necessary for a specific biomedical study
  - Yes
  - No

Name participant:
Date of birth:
Signature: Date: __/ __/ __

Declaration of researcher

I hereby declare that I have fully informed the legal representative regarding the biobank Pearl AAA. If information comes to light during the course of the study that could affect the legal representative's consent, I will inform him/her of this in a timely fashion.

Name researcher:
Signature: Date: __/ __/ __

Additional information has been given by (if applicable):
Name:
Role:
Signature: Date: __/ __/ __

Study participants receive the full information brochure, and a signed copy of the informed consent form.

For peer review only

English translation of the informed consent form of the PARIS study

Appendix 4. Informed consent form for patients after emergency AAA repair.

PARIS study

- I have read the subject information form. I was also able to ask questions. My questions have been answered to my satisfaction. I had enough time to decide whether to participate.
- I know that participation is voluntary. I know that I may decide at any time not to participate after all or to withdraw from the study. I do not need to give a reason for this.
- I give permission for my GP to be informed that I am participating in this study.
- I give permission for information to be requested from my GP or pharmacy regarding my health status.
- I know that some people may have access to all my data to verify the study. These people are listed in this information sheet. I consent to the inspection by them.
- I consent to the collection and use of my data, biomaterials and imaging data as has been specified in this information sheet.
- I consent to the storage of my data for the PARIS study for a maximum of 50 years in the biobank Pearl AAA.
- I consent to the possible sharing of my data, biomaterials and imaging data with non-commercial institutions in countries in the European Union. This will only be done for the PARIS study if necessary.

Additional consent options:

- I consent to the possible sharing of my data, biomaterials and imaging data with institutions in countries outside the European Union. This will only be done for the PARIS study if necessary.
    - Yes
    - No
- I consent to the possible sharing of my data, biomaterials and imaging data with commercial entities. This will only be done for the PARIS study if necessary.
    - Yes
    - No
- I consent to being contacted again after this study for a follow-up study.
    - Yes
    - No
- I want to participate in this study.

Name participant:
Date of birth:

For peer review only

English translation of the informed consent form of the PARIS study

Signature: Date: __/ __/ __

Declaration of researcher

I hereby declare that I have fully informed this study participant regarding the PARIS study. If information comes to light during the course of the study that could affect the study subject's consent, I will inform him/her of this in a timely fashion.

Name researcher:
Signature: Date: __/ __/ __

Additional information has been given by (if applicable):
Name:
Role:
Signature: Date: __/ __/ __

Study participants receive the full information brochure, and a signed copy of the informed consent form.

For peer review only

English translation of the informed consent form of the biobank Pearl AAA

Appendix 5. Informed consent form for legal representative.

Biobank Pearl AAA

I have been asked to give written consent for the participation of the following person in the biobank Parel AAA.

Name person:
Date of birth: __/ __/ __

- I consent to the collection, storage and analysis of blood, urine and aortic tissue (in case of open surgery) and data of the person regarding research for abdominal aortic aneurysms.

- I consent to the coded storage of the data for a maximum of 50 years in the biobank Pearl AAA.

- I consent to the coded storage of the biomaterials for a maximum of 50 years for use in future biomedical research.

- I give permission that the GP of the person is to be informed that the person is participating in this study.

- I give permission for information regarding the health status of the person to be requested from the GP and pharmacy, under the condition that his/her privacy will be safeguarded.

- During biomedical research with the biomaterials, incidental findings may be found that are associated with a disease or disorder. Sometimes it only concerns an increased risk. I consent that the GP of the person will be informed about incidental findings if these are relevant to the family's health status because either prevention or treatment is possible.

- I consent to the possible sharing of the data, biomaterials and imaging data with non-commercial institutions in countries the European Union. This will only be done for research purposes.

Additional consent options:

- I consent to the acquisition of data regarding the person from national registries concerning the quality of healthcare and its improvement, under the condition that his/her privacy will be safeguarded.
  - Yes
  - No

- I consent to the possible sharing of the data, biomaterials and imaging data of the person with institutions in countries outside the European Union. This will only be done for research purposes.
  - Yes
  - No

- I consent to the possible sharing of the data, biomaterials and imaging data with commercial entities. This will only be done for research purposes.

For peer review only

English translation of the informed consent form of the biobank Pearl AAA

- Yes
- No

- I consent to the study of the DNA for findings associated with the origin and development of abdominal aortic aneurysms and to the effects and complications of medication
  - Yes
  - No

Name legal representative:
Date of birth:
Relation to the person:
Signature: Date: __/ __/ __

Declaration of researcher

I hereby declare that I have fully informed the legal representative regarding the biobank Pearl AAA. If information comes to light during the course of the study that could affect the legal representative's consent, I will inform him/her of this in a timely fashion.

Name researcher:
Signature: Date: __/ __/ __

Additional information has been given by (if applicable):
Name:
Role:
Signature: Date: __/ __/ __

Study participants receive the full information brochure, and a signed copy of the informed consent form.

For peer review only

English translation of the informed consent form of the PARIS study

Appendix 6. Informed consent form for legal representative.

PARIS study

I have been asked to give written consent for the participation of the following person in the PARIS study.

Name person:
Date of birth: __/ __/ __

- I have read the subject information form. I was also able to ask questions. My questions have been answered to my satisfaction. I had enough time to decide whether this person participates.
- I know that participation is voluntary. I know that I may decide at any time that this person does not to participate after all or to withdraw him/her from the study. I do not need to give a reason for this.
- I give permission for the GP of this person to be informed that he/she is participating in this study.
- I give permission for information to be requested from the GP or pharmacy of this person regarding his/her health status.
- I know that some people may have access to all this person's data to verify the study. These people are listed in this information sheet. I consent to the inspection by them.
- I consent to the collection and use of the data, biomaterials and imaging data as has been specified in this information sheet.
- I consent to the storage of the data for the PARIS study for a maximum of 50 years in the biobank Pearl AAA.
- I consent to the possible sharing of the data, biomaterials and imaging data with non-commercial institutions in countries in the European Union. This will only be for the PARIS study if necessary.

- I consent to the possible sharing of the data, biomaterials and imaging data with institutions in countries outside the European Union. This will only be done for the PARIS study.
    - Yes
    - No
- I consent to the possible sharing of the data, biomaterials and imaging data with commercial entities. This will only be done for the PARIS study.
    - Yes
    - No
- I agree with the participation of this person with the study.

For peer review only

English translation of the informed consent form of the PARIS study

Name legal representative:
Date of birth:
Relation to the person:
Signature: Date: __/ __/ __

Declaration of researcher

I hereby declare that I have fully informed the legal representative regarding the biobank Pearl AAA. If information comes to light during the course of the study that could affect the legal representative's consent, I will inform him/her of this in a timely fashion.

Name researcher:
Signature: Date: __/ __/ __

Additional information has been given by (if applicable):
Name:
Role:
Signature: Date: __/ __/ __

Study participants receive the full information brochure, and a signed copy of the informed consent form.

For peer review only
